# When do correlations increase with firing rates in recurrent networks?

**DOI:** 10.1371/journal.pcbi.1005506

**Published:** 2017-04-27

**Authors:** Andrea K. Barreiro, Cheng Ly

**Affiliations:** 1 Department of Mathematics, Southern Methodist University, Dallas, Texas, United States of America; 2 Department of Statistical Sciences and Operations Research, Virginia Commonwealth University, Richmond, Virginia, United States of America; University College London, UNITED KINGDOM

## Abstract

A central question in neuroscience is to understand how noisy firing patterns are used to transmit information. Because neural spiking is noisy, spiking patterns are often quantified via pairwise correlations, or the probability that two cells will spike coincidentally, above and beyond their baseline firing rate. One observation frequently made in experiments, is that correlations can increase systematically with firing rate. Theoretical studies have determined that stimulus-dependent correlations that increase with firing rate can have beneficial effects on information coding; however, we still have an incomplete understanding of what circuit mechanisms do, or do not, produce this correlation-firing rate relationship. Here, we studied the relationship between pairwise correlations and firing rates in recurrently coupled excitatory-inhibitory spiking networks with conductance-based synapses. We found that with stronger excitatory coupling, a positive relationship emerged between pairwise correlations and firing rates. To explain these findings, we used linear response theory to predict the full correlation matrix and to decompose correlations in terms of graph motifs. We then used this decomposition to explain why covariation of correlations with firing rate—a relationship previously explained in feedforward networks driven by correlated input—emerges in some recurrent networks but not in others. Furthermore, when correlations covary with firing rate, this relationship is reflected in low-rank structure in the correlation matrix.

## Author summary

A central question in neuroscience is to understand how noisy firing patterns are used to transmit information. We quantify spiking patterns by using pairwise *correlations*, or the probability that two cells will spike coincidentally, above and beyond their baseline firing rate. One observation frequently made in experiments is that correlations can increase systematically with firing rate. Recent studies of a type of output cell in mouse retina found this relationship; furthermore, they determined that the increase of correlation with firing rate helped the cells encode information, provided the correlations were stimulus-dependent. Several theoretical studies have explored this basic structure, and found that it is generally beneficial to modulate correlations in this way. However—aside from mouse retinal cells referenced here—we do not yet have many examples of real neural circuits that show this correlation-firing rate pattern, so we do not know what common features (or mechanisms) might occur between them.

In this study, we address this question via a computational model. We set up a computational model with features representative of a generic cortical network, to see whether correlations would increase with firing rate. To produce different firing patterns, we varied excitatory coupling. We found that with stronger excitatory coupling, there was a positive relationship between pairwise correlations and firing rates. We used a network linear response theory to show why correlations could increase with firing rates in some networks, but not in others; this could be explained by how cells responded to fluctuations in inhibitory conductances.

## Introduction

One prominent goal of modern theoretical neuroscience is to understand how the features of cortical neural networks lead to modulation of spiking statistics [1–3]. This understanding is essential to the larger question of how sensory information is encoded and transmitted, because such statistics are known to impact population coding [4–8]. Both experimental and theoretical inquiries are complicated by the fact that neurons are widely known to have heterogeneous attributes [9–14].

One family of statistics that is implicated in nearly all population coding studies is trial-to-trial variability (and co-variability) in spike counts; there is now a rich history of studying how these statistics arise, and how they effect coding of stimuli [15–19]. Recent work by numerous authors has demonstrated that the information content of spiking neural activity depends on spike count correlations and its relationship (if any) with stimulus tuning [15, 17, 19–21]. Since a population of sensory neurons might change their firing rates in different ways to stimuli, uncovering the general mechanisms for when spiking correlations increases with firing rate (or when they do not) is important in the context of neural coding. Thus, we study this question in a general recurrent neural network model.

One observation that has been made in some, but not all, experimental studies is that pairwise correlations increase with firing rates. This relationship has been observed *in vitro* [22] and in several visual areas: area MT [23], V4 [24], V1 [25, 26], and notably, in ON-OFF directionally sensitive retinal ganglion cells [21, 27]. The retinal studies involved cells with a clearly identified function, and therefore allowed study of the coding consequences of this correlation/firing rate relationship. Both studies found that the *stimulus-dependent* correlation structure observed compared favorably to a structure in which *stimulus-independent* correlations were matched to their (stimulus-)averaged levels. This finding reflects a general principle articulated in other studies [17, 19], that stimulus-dependent correlations are beneficial when they serve to spread the neural response in a direction *orthogonal* to the signal space.

While many studies have illustrated the connection between stimulus-dependent correlation structure and coding, these have (until recently: see [21, 25, 27]) largely taken the correlation structure as given, leaving open the question of how exactly a network might produce the hypothesized correlation structure [6, 7] (see also the theoretical calculations in [21, 27]). Theoretical studies of the *mechanisms* that contribute to correlation distributions have largely analyzed homogeneous networks (i.e. cells are identical, aside from E/I identity) [2, 3, 28, 29], which does not allow an exploration of a correlation/firing rate relationship. Thus, how correlation coefficients can vary across a population of heterogeneously-tuned neurons is not yet well understood despite its possible implications for coding.

In this paper we investigated the relationship between correlations and firing rates in conductance-based leaky integrate-and-fire (**LIF**) neural network models, consisting of excitatory (E) and inhibitory (I) cells that are recurrently and randomly coupled. We introduced neural heterogeneity by allowing thresholds to vary across the population, which induced a wide range of firing rates, and explored different firing regimes by varying the strength of recurrent excitation. We found that with relatively strong excitation, pairwise correlations increased with firing rate.

In theoretical studies, this correlation-firing rate trend has been explained in feed-forward networks driven by common input [22, 30, 31]. Here we investigated whether the correlation/firing relationship in recurrent networks can be explained by this theory, but where the source of input correlations is internally generated; i.e., from overlapping projections within the recurrent network. We first adapted a network linear response theory, to decompose predicted correlations into contributions from different graph motifs, which are subgraphs which form the building blocks of complex networks [28, 32, 33].

We found that in all networks studied here, second-order motifs—and specifically *inhibitory common input*—were the dominant contributor to overall pairwise correlations. This allowed us to generalize theory from [22], and describe pairwise correlations in terms of a single-cell susceptibility function. Surprisingly, we found that correlations from inhibitory common input could either increase *or* decrease with firing rate, depending on how cells responded to fluctuations in inhibitory conductances.

We further show that a correlation-firing rate relationship has an important consequence for heterogeneous networks; it can shape low-dimensional structure in the correlation matrix. Low-dimensional structure—often modeled with a low-rank approximation to the correlation matrix—is important because it can be used to improve estimation [34] and even to reconstruct full correlation matrices from incomplete data [35–37]; such structure has been observed in experimental data [25, 38–41] but its origin is not always known. We demonstrate in our networks that when correlation co-varies with firing rate, the (E-E) correlation matrix could be accurately modeled with a low-rank approximation, and the low-rank projection in this approximation was strongly associated with firing rate. Thus we demonstrate that low-rank structure can result from recurrent activity modulated by single-cell characteristics, as well as from a global input or a top-down signal [38].

## Results

We studied asynchronous recurrent networks of leaky integrate-and-fire model neurons, and varied the strength of excitation to get different firing behaviors. We found that the covariation of correlations with firing rates—a phenomenon observed in feed-forward networks—occurs here in one firing regime, but not the other. We then found that this could be explained in terms of how single cells responded to fluctuations in inhibitory conductance. Finally, we show that when correlations covary with firing rates, the correlation matrix admits a low-rank approximation.

### Asynchronous firing in heterogeneous networks

**Fig 1:**
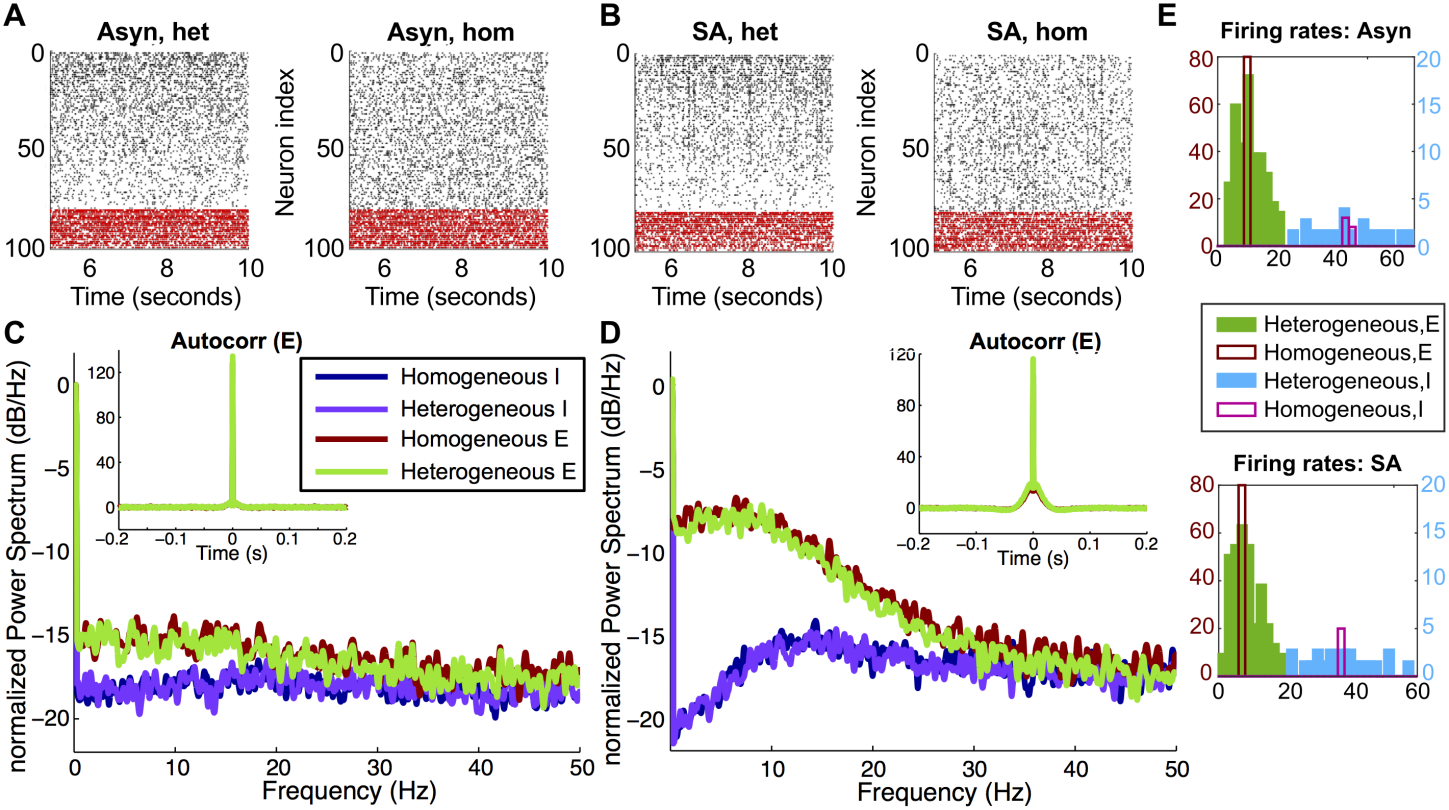
Two firing regimes in heterogeneous networks. Monte Carlo simulations illustrating two firing regimes we consider in this paper. (A) Raster plots from the asynchronous (**Asyn**) regime. (B) Raster plots from the *strong asynchronous* (**SA**) regime, showing occasional bursts of activity. (C) Power spectra in the asynchronous regime. (D) Power spectra in the strong asynchronous regime. (E) Firing rates in the asynchronous (top panel) and SA (bottom panel) regimes. In (A-B), cells are ordered by increasing threshold value. Power spectra (C-D) are normalized to their maximum value and expressed in decibels/Hz.

We performed Monte Carlo simulations of recurrent, randomly connected E/I networks, as described in **Methods: Neuron model and network setup**. To connect to previous literature on asynchronous spiking, we compared networks with and without single-cell variability—referred to as *heterogeneous* and *homogeneous* respectively. Heterogeneity was introduced by allowing cell threshold to vary, which induced a corresponding range of firing rates (see Methods**: Neuron model and network setup** for details). We first chose parameters so that the networks exhibited the classical *asynchronous irregular* (**Asyn**) regime, in which each neuron has irregular Poisson-like spiking, correlations are low, and the population power spectra are flat [42]. In Fig 1A we show raster plots from both the heterogeneous and homogeneous networks, in this regime. The heterogeneous network shows a gradient in its raster plot, because cells are ordered by decreasing firing rate. The population power spectra were flat, for both E and I cells and in both homogeneous and heterogeneous networks (Fig 1C).

When we increased excitation (by increasing both *W*_*EE*_ and *W*_*IE*_, where *W*_*XY*_ is the conductance strength from type *Y* to *X*; see Table 1 for parameter values), we observed occasional bursts of activity. However, the bursts do not occur at regular intervals and do not involve the entire population (we found excitatory bursts involved at most 25% of the population). The network is still moderately inhibition-dominated and neurons are spiking irregularly; example raster plots are shown in Fig 1B. The population power spectra (Fig 1D) are no longer flat (compare to the asynchronous regime, Fig 1C); they show local maxima around 8 Hz, but it is not a pronounced peak. We will refer to this as the *strong asynchronous* (**SA**) regime [43].

**Table 1:** Excitatory connection strengths mediate between different firing regimes.

Parameter	*W*_*EI*_ (*I* → *E*)	*W*_*IE*_ (*E* → *I*)	*W*_*EE*_	*W*_*II*_	*σ*_*i*_(*i* ∈ *E*)	*σ*_*i*_(*i* ∈ *I*)
Asynchronous Str. Asynch.	10 10	5 8	0.5 9	5 5	$2/\sqrt{2}$ $1.5/\sqrt{2}$	$3/\sqrt{2}$ $2.5/\sqrt{2}$
% connectivity	35%	20%	40%	40%		

Here *W*^*YX*^ denotes *X* → *Y* connections; e.g. *W*^*IE*^ denotes the strength of excitatory connections onto inhibitory neurons. The parameter *σ*_*i*_ denotes the strength of background noise in units of (scaled) voltage, and depends only on cell type (*E* or *I*).

In both Fig 1C and 1D, we note that—despite the apparent differences in the distribution of spikes across the network, evident in the raster plots—both the autocorrelation functions (Fig 1C and 1D, insets) and the power spectra from the heterogeneous and homogeneous networks are very similar. Thus, we have a fair comparison to examine the role of heterogeneity, independent of other characteristics of the network.

The distribution of both excitatory and inhibitory firing rates are extremely narrow in the homogeneous network, but broad in the heterogeneous network (Fig 1E). This is expected, as each excitatory (inhibitory) cell in the homogenous network has the same uncoupled firing rate; because the number of synaptic inputs is likewise fixed, population variability in synaptic input is limited. The heterogeneous networks have a range of firing rates, which allows us to investigate the possibility of a relationship between (variable) firing rate and pairwise correlations. Population-averaged firing rates were very similar between the heterogeneous and homogeneous networks: in the asynchronous regime ⟪*ν*_*E*_⟫ = 10.6 Hz (heterogeneous) and ⟪*ν*_*E*_⟫ = 10.1 Hz (homogeneous), while ⟪*ν*_*I*_⟫ = 44.3 Hz (heterogeneous) and ⟪*ν*_*I*_⟫ = 43.5 Hz (homogeneous). In both regimes Fano factors ranged between 0.9 and 1.1, consistent with Poisson-like spiking (more statistics are given in S1 and S3 Tables).

### Correlation increases with firing rate in the strong asynchronous regime

We next sought a possible relationship between pairwise correlations—quantified via the Pearson’s correlation coefficient for spike counts, $\rho_{ij}\equiv {\mathrm{Cov}}_{T}\left(n_{i},n_{j}\right)/\sqrt{{\mathrm{Var}}_{T}\left(n_{i}\right){\mathrm{Var}}_{T}\left(n_{j}\right)}$—and single-cell firing rates. Such relationships have been found in feed-forward networks [22, 30, 31], and impact information transfer when considered in concert with stimulus selectivity (i.e. signal correlations) [7, 8, 15, 19]. In heterogeneous networks, the large range of firing rates—equivalently the large range of operating points—admits the possibility that cells at different operating points may differ in their ability to transfer correlations.

**Fig 2:**
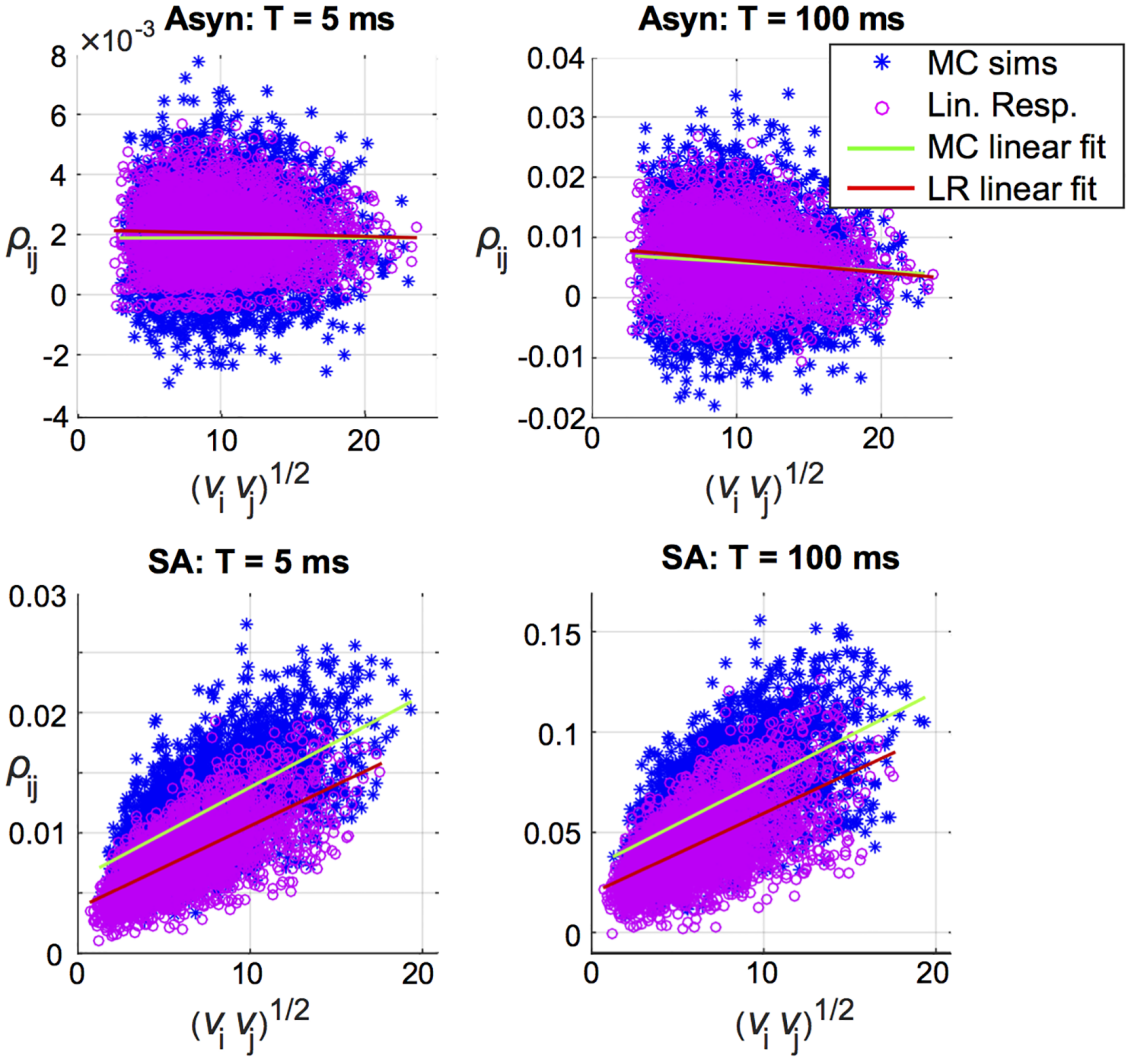
Correlation increases with firing rate in the strong asynchronous regime. E-E correlation *ρ*_*ij*_ vs. geometric mean firing rate $\sqrt{\nu_{i}\nu_{j}}$, cell-by-cell comparison of Monte Carlo simulations (blue stars) and linear response (magenta circles), in a heterogeneous network. Left to right: time window *T* = 5 ms and 100 ms. Top row: asynchronous regime. Bottom row: strong asynchrony.

To investigate this we plotted pairwise correlations for each distinct excitatory pair *ρ*_*ij*_, versus the geometric mean of the firing rates $\sqrt{\nu_{i}\nu_{j}}$, in both regimes (asynchronous and strong asynchronous), for a range of time scales (blue stars in Fig 2). We focus here on excitatory-excitatory (E-E) pairs, because excitatory synaptic connections provide the predominant means of propagating cortical sensory information to higher layers. Our results show a striking difference between the two spiking regimes; while there is no clear relationship with firing rate in the asynchronous regime (Fig 2, top row), the strong asynchronous regime shows a distinct positive trend with firing rate (Fig 2, bottom row). We can quantify a hypothesized relationship between *ν* and *ρ* with linear regression, and indeed find that geometric mean firing rate explains a substantial part of the variability of correlations in the strong asynchronous regime obtained from the Monte Carlo simulations, with *R*^2^ values (i.e. percentage of variability explained) of 0.41, 0.37, and 0.34 for time windows of *T* = 5, 50, and 100 ms respectively (in contrast, *R*^2^ values for the asynchronous network are below 0.005).

In recurrent networks, the response of each cell is shaped by both direct and indirect connections through the network. We used the linear response theory described in **Methods: Linear Response Theory** and **Methods: Computing statistics from linear response theory** to predict the full correlation matrix **C**_*T*_ at various time scales, including the limit of long time scales: $\tilde{C}\left(0\right)=\lim_{T\to \infty }\frac{1}{T}C_{T}$. We found that this theory successfully captured E-E correlations, both the full distribution of values and coefficients of individual cell pairs (details, including figures, can be found in: **Supporting Information:** S1 Text).

We then plotted the predicted correlation, ${\tilde{C}}_{ij}/\sqrt{{\tilde{C}}_{ii}{\tilde{C}}_{jj}}$, vs. geometric mean firing rate $\sqrt{\nu_{i}\nu_{j}}$ (magenta circles in Fig 2). The predicted correlations captured the same positive relationship observed in Monte Carlo results, with *R*^2^ values of 0.47, 0.4, and 0.36.

### Decomposition of correlation by graph motifs shows strong role for second-order motifs

**Fig 3:**
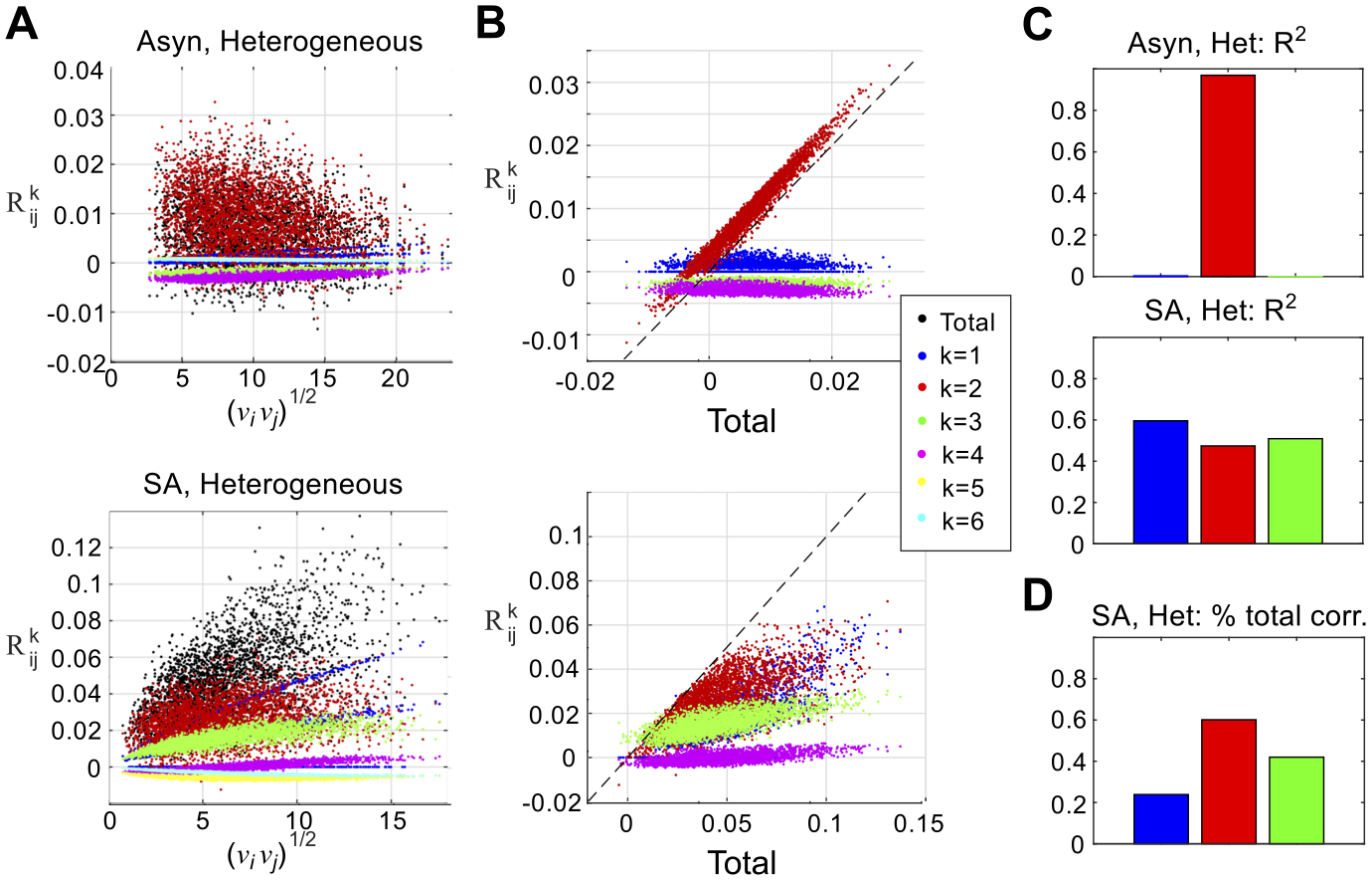
Pairwise correlations are built from graph motifs. Contributions of different orders to prediction of E-E correlations with linear response theory. (A) Normalized contributions to pairwise correlation (${\tilde{R}}_{ij}^{k}$) vs. geometric mean firing rate ($\sqrt{\nu_{i}\nu_{j}}$) for heterogeneous networks in the asynchronous (top panel) and strong asynchronous (bottom panel) regimes. (B) As in (A), but plotted vs. total predicted normalized correlation (${\tilde{C}}_{ij}/\sqrt{{\tilde{C}}_{ii}{\tilde{C}}_{jj}}$). See main text for further discussion. (C) To quantify the relative importance of different motifs, we report the fraction of variance explained (*R*^2^) from linear regressions, in which we regressed total correlation (${\tilde{C}}_{ij}/\sqrt{{\tilde{C}}_{ii}{\tilde{C}}_{jj}}$) against the contributions at each specific order (${\tilde{R}}_{ij}^{k}$). As suggested by (B), second-order contributions (red) overwhelmingly determine total correlations in the asynchronous network (*R*^2^ values for first- and third-order terms are shown, but barely visually distinguishable; *R*^2^ values for higher orders are also small, within 0.08 up to *k* = 6). (D) Fraction of total correlation from each order, strong asynchronous regime.

Why does a correlation/firing rate relationship emerge in one spiking regime, but not the other? In feed-forward networks, a positive correlation/firing rate relationship results from transferring common input through fluctuation-driven, asynchronously-firing cells [22, 30]. In contrast, the amount of shared input into two cells in a recurrent network is determined by both direct and indirect connections through the network. To separate the impact of different network pathways, we decomposed the *linear response-predicted* correlations at long time scales (i.e. $\tilde{C}\left(0\right)=\lim_{T\to \infty }\frac{1}{T}C_{T}$) into normalized contributions from *n*-th order motifs, as described in **Methods: Quantifying the role of motifs in networks**. Common input from a divergent connection, for example, results from the 2nd-order motif K*C^0^**K**. In Fig 3, we plot the summed contributions up to sixth order—i.e. ${\tilde{R}}_{ij}^{k}$, for *k* = 1, 2, …6—versus geometric mean firing rate, $\sqrt{\nu_{i}\nu_{j}}$. The total normalized correlation, ${\tilde{C}}_{ij}/\sqrt{{\tilde{C}}_{ii}{\tilde{C}}_{jj}}$, is shown as well. In all cases, we plot long time scale correlations *ω* = 0; each distinct E-E pair is represented.

In the asynchronous regime (top panel of Fig 3A), first-order contributions (${\tilde{R}}^{1}$) separate into three distinct “curves”, reflecting a 1-1 relationship with firing rate conditioned on first-order connectivity (no connection between *i* and *j*; one connection between *i* and *j*; bidirectional connection between *i* and *j*). Second-order contributions are overall positive while third-order contributions are overall negative (consistent with [28]); neither appear to have a relationship with firing rate. Second-order contributions are conspicuously dominant; fifth and sixth order terms are near zero.

This qualitative picture changes when we consider the strong asynchronous regime (bottom panel of Fig 3A). First-order contributions follow a similar pattern as in the asynchronous regime, and second-order contributions are likewise positive. However, third-order contributions are positive, and in the heterogeneous network they have a distinctly positive relationship with firing rate (top panel). Thus, in the asynchronous regime, negative third-order contributions partially cancel with positive second-order contributions; in the strong asynchronous regime, first, second, and third-order motifs reinforce each other, contributing to an overall positive relationship with firing rate (black dots).

Despite these differences, second-order contributions are the major determinant of total correlation in both regimes. In Fig 3B we plot the same data (${\tilde{R}}_{ij}^{k}$) vs. total correlation, rather than geometric mean firing rate. In the asynchronous regime, second-order contributions cluster near the unity line, suggesting they are strongly predictive of total correlation. To quantify this intuition we computed the fraction of variance explained (*R*^2^) by performing a linear regression of total normalized correlation (${\tilde{C}}_{ij}/\sqrt{{\tilde{C}}_{ii}{\tilde{C}}_{jj}}$) against contributions of each order (Fig 3C); in the asynchronous regime *R*^2^ values for ${\tilde{R}}_{ij}^{1}$, ${\tilde{R}}_{ij}^{2}$, and ${\tilde{R}}_{ij}^{3}$ were 0.004, 0.969, and 0.0002, respectively. *R*^2^ values for higher orders were likewise small: for ${\tilde{R}}_{ij}^{4}$, ${\tilde{R}}_{ij}^{5}$, and ${\tilde{R}}_{ij}^{6}$ they were 0.047, 0.034, and 0.074.

This statistic was more ambiguous in the strong asynchronous regime, where *R*^2^ values for ${\tilde{R}}_{ij}^{1}$, ${\tilde{R}}_{ij}^{2}$, and ${\tilde{R}}_{ij}^{3}$ were 0.595, 0.474, and 0.509 respectively. However, note that ${\tilde{R}}_{ij}^{1}$ and ${\tilde{R}}_{ij}^{3}$ were positive for all cell pairs; ${\tilde{R}}_{ij}^{2}$ and total correlation were negative for less than 0.3% of cell pairs. Thus, we considered how each motif contributed to the total correlation by taking the ratio of each contribution to the total, averaged over all cell pairs (Fig 3D). By this measure, second-order contributions were largest; fraction explained for ${\tilde{R}}_{ij}^{1}$, ${\tilde{R}}_{ij}^{2}$, and ${\tilde{R}}_{ij}^{3}$ were 0.239, 0.601, and 0.420, respectively. Note that this measure cannot be used for the asynchronous (**Asyn**) regime because of the negative values of ${\tilde{R}}_{ij}^{k}$.

Taken together, this evidence points to a distinguished role for second-order motifs (${\tilde{R}}_{ij}^{2}$) in determining total correlation. In the asynchronous regime in particular, ${\tilde{R}}_{ij}^{2}$ is a near-perfect predictor of total correlation.

### Inhibitory common input is the most important second-order motif

**Fig 4:**
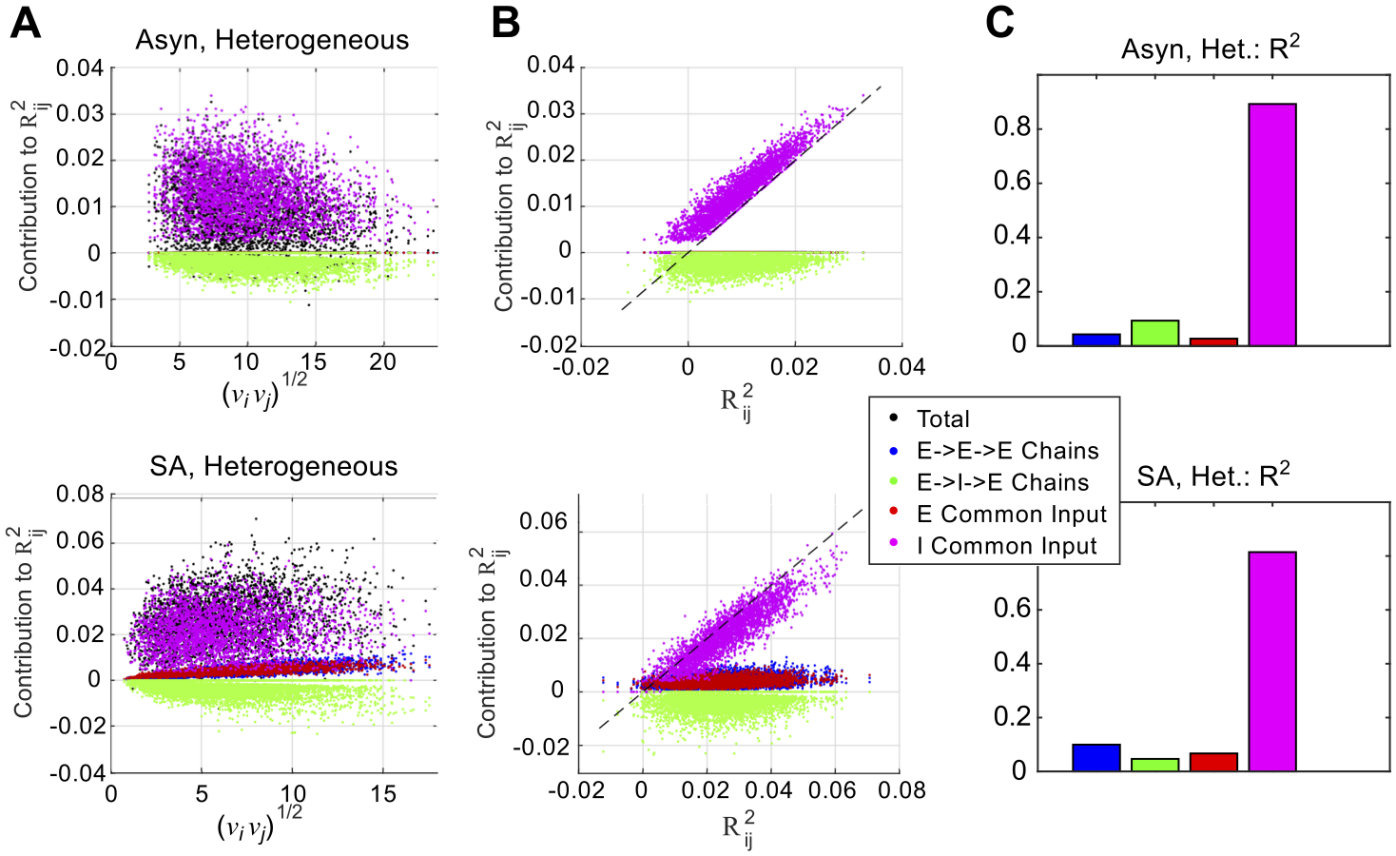
Inhibitory common input is the dominant second-order motif in both asynchronous and strong asynchronous networks. (A) Contributions of different 2nd-order motifs to prediction of E-E correlations in a heterogeneous network, in the asynchronous (top) and strong asynchronous (bottom) regimes. (B) As in (A), but plotted vs. total contribution from second-order motifs ${\tilde{R}}^{2}$. In both panels, inhibitory common input (magenta) clusters near the unity line. (C) To quantify the relative importance of different motifs, we report the fraction of variance explained (*R*^2^) from linear regressions, in which we regressed the total contribution from second-order motifs (${\tilde{R}}_{ij}^{k}$) against the contribution from specific motifs types.

We next analyzed contributions from specific **second-order motifs** in Fig 4. There are four distinct second-order motifs that can correlate two E cells. There are two types of chains, from **K**^2^**C**^0^ and **C**^0^ (K*)^2^. An *E* → *E* → *E* chain tends to positively correlate, while an *E* → *I* → *E* chain will negatively correlate; these are shown as blue and green respectively. There are two types of common input, from **KC**^0^(K*); they correspond to common input from E and I cells, i.e. *E* ← *E* → *E* and *E* ← *I* → *E*. They *both* lead to positive correlations and are shown as red and magenta respectively.

In the asynchronous regime (left panel of Fig 4A), the dominant contributions are *I* common input (magenta) and negative (*E* → *I* → *E*) chains (green); correlating chains (blue) and excitatory common input (red) are barely visible, as they are clustered near zero. In the strong asynchronous case (right panel), blue and red dots are now visible and show a clear 1-1 trend with firing rate. In both regimes, *inhibitory common input* appears to be the dominant second-order motif. In Fig 4B we plot the contribution from different second-order motifs vs. the total contribution from second-order motifs, ${\tilde{R}}_{ij}^{2}$ (rather than geometric mean firing rate, $\sqrt{\nu_{i}\nu_{j}}$). In both panels, the inhibitory common input (magenta) clusters around the unity line, showing it is the best predictor of the total second-order contribution. In Fig 4C we quantify this observation by reporting fraction of variance explained (*R*^2^) from linear regressions: the *R*^2^ value for inhibitory common input exceeds 0.8 in both networks, while the *R*^2^ values for all other motifs types are less than 0.1.

In conclusion, decomposition of pairwise correlations into graph motifs has shown us two important things: first, while third-order motifs probably contribute to the positive correlation/firing rate relationship observed in the SA regime, second-order motifs still dominate in both regimes. Second, **inhibitory common input is the most important second-order motif in both regimes** (Fig 4B).

### Susceptibility to inhibition can either increase or decrease with firing rate

In feedforward networks—i.e., in the absence of a path between two cells—correlations in *outputs* (i.e. spike trains) must arise from correlations in *inputs*; for example, through shared or common inputs. We have found that inhibitory common input is the dominant contributor to pairwise correlations in both the asynchronous and strong asynchronous regimes; we now turn our attention to modeling this term (inhibitory common input) specifically.

Previous work that analyzed the relationship between the long-time correlation and firing rate in feedforward networks [22, 30] quantified a *susceptibility* function that measures the ratio between output and input correlations:

$$S\approx \frac{\rho }{c}.$$ (1)

If both cells receive a large (but equal) number of uncorrelated inputs, *c* would be the fraction of inputs that are common to both *i* and *j*.

In the networks examined here, each cell had a fixed in-degree for both excitatory and inhibitory cells; however, for any given *pair* of cells *i* and *j*, the number of E and I inputs that synapsed onto both cells will vary from pair to pair. Thus, we next considered the possibility that our (negative) finding in the asynchronous network could be explained by accounting for variable *c*_*ij*_.

**Fig 5:**
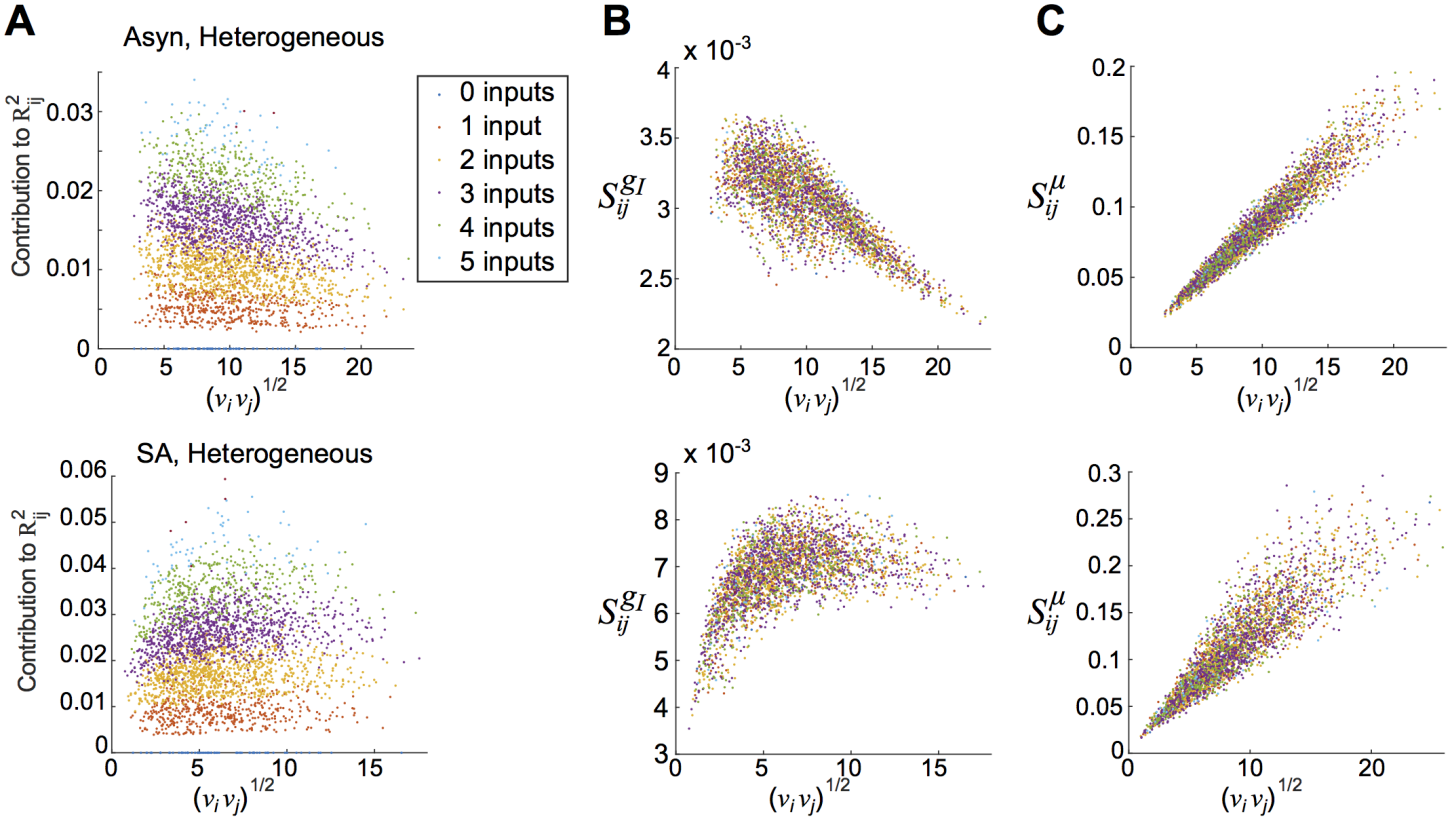
Susceptibility to conductance fluctuations can explain correlation-firing rate relationships. In (A-C): heterogeneous asynchronous (top) and heterogeneous strong asynchronous (bottom). (A) Correlation (*ρ*) from I common inputs vs. firing rate, segregated based on the number of common inhibitory inputs. (B) Estimated correlation susceptibility to fluctuations in inhibitory *conductances* vs. firing rate ($S_{ij}^{g_{I}}$). (C) Correlation susceptibility to fluctuations in inhibitory *currents* vs. firing rate ($S_{ij}^{\mu }$).

We focus on inhibitory common input, which is the dominant second-order contribution in the asynchronous network (Fig 4). We segregated pairs by whether they had 0, 1, 2, etc.. common inhibitory inputs; we then use this number as a proxy for *c* (recall that each excitatory cell had exactly 7 inhibitory inputs, so that this number divided by 7 approximates the common input fraction; two common inputs imply *c* ≈ 0.28 for example). We plot the results for the asynchronous network in Fig 5A, top panel (data for each distinct value of *c* is presented by color). As we might expect, correlation increases as *c* increases. However, for a fixed *c*, there is not an apparent relationship between firing rate and correlation; if anything, there appears to be a slight decrease. Correlation also increases with *c* in the strong asynchronous network (Fig 5A, bottom panel); however, here we also see a modest increase with geometric mean firing rate $\sqrt{\nu_{i}\nu_{j}}$.

Previous theoretical work [22, 30] identified an increase in susceptibility with firing rates in *current-driven* neurons; we next considered the possibility that this fails to hold for conductance-driven neurons. As described in **Methods: Quantifying correlation susceptibility**, we estimated correlation susceptibility for each pair of neurons, by using the susceptibility function for each neuron to conductance fluctuations (computed as part of the linear response theory), divided by a measure of the long-timescale spike count variance:

$$S_{ij}^{\left\langle g_{I}\right\rangle }=\frac{{\tilde{A}}_{\langle g_{I}\rangle ,i}\left(0\right){\tilde{A}}_{\langle g_{I}\rangle ,j}\left(0\right)}{\sqrt{{\tilde{C}}_{ii}\left(0\right){\tilde{C}}_{jj}\left(0\right)}}$$ (2)

We plotted the results for both networks in Fig 5B; while susceptibility increases with firing rate in the strong asynchronous network (except for the largest firing rates), it actually decreases with firing rate in the asynchronous network.

We can contrast with the estimated susceptibility to *current* fluctuations (i.e. *A*_*μ*,*i*_, with *μ*_*i*_, *τ*_eff,*i*_, and *σ*_eff,*i*_ as in Eq 29) which we also computed for the same set of cell pairs, shown in Fig 5C.

$$S_{ij}^{\mu }=\frac{{\tilde{A}}_{\mu ,i}\left(0\right){\tilde{A}}_{\mu ,j}\left(0\right)}{\sqrt{{\tilde{C}}_{ii}\left(0\right){\tilde{C}}_{jj}\left(0\right)}}$$ (3)

Here, we see that $S_{ij}^{\mu }$ increases with firing rates, in both networks.

### Understanding the susceptibility function

We next sought to understand how susceptibility depends on neural parameters; that is, we define the single-cell susceptibility

$$S_{i}^{\left\langle g_{I}\right\rangle }\equiv \frac{{\tilde{A}}_{\langle g_{I}\rangle ,i}\left(0\right)}{\sqrt{\nu_{i}}}$$ (4)

where

$$\nu_{i}=f\left(\left\langle g_{I,i}\right\rangle ,\sigma_{I,i},\left\langle g_{E,i}\right\rangle ,\sigma_{E,i},\sigma_{i},\theta_{i}\right)$$ (5)

$${\tilde{A}}_{\langle g_{I}\rangle ,i}\left(0\right)=\frac{\partial f}{\partial x_{1}}\left(\left\langle g_{I,i}\right\rangle ,\sigma_{I,i},\left\langle g_{E,i}\right\rangle ,\sigma_{E,i},\sigma_{i},\theta_{i}\right).$$ (6)

(“$\frac{\partial }{\partial x_{1}}$” indicates that derivative is taken with respect to the first argument, 〈*g*_*I*, *i*_〉). We have also used the asynchronous spiking assumption, that ${\tilde{C}}_{ii}\left(0\right)\approx \nu_{i}$ (compare with Eq 3).

**Fig 6:**
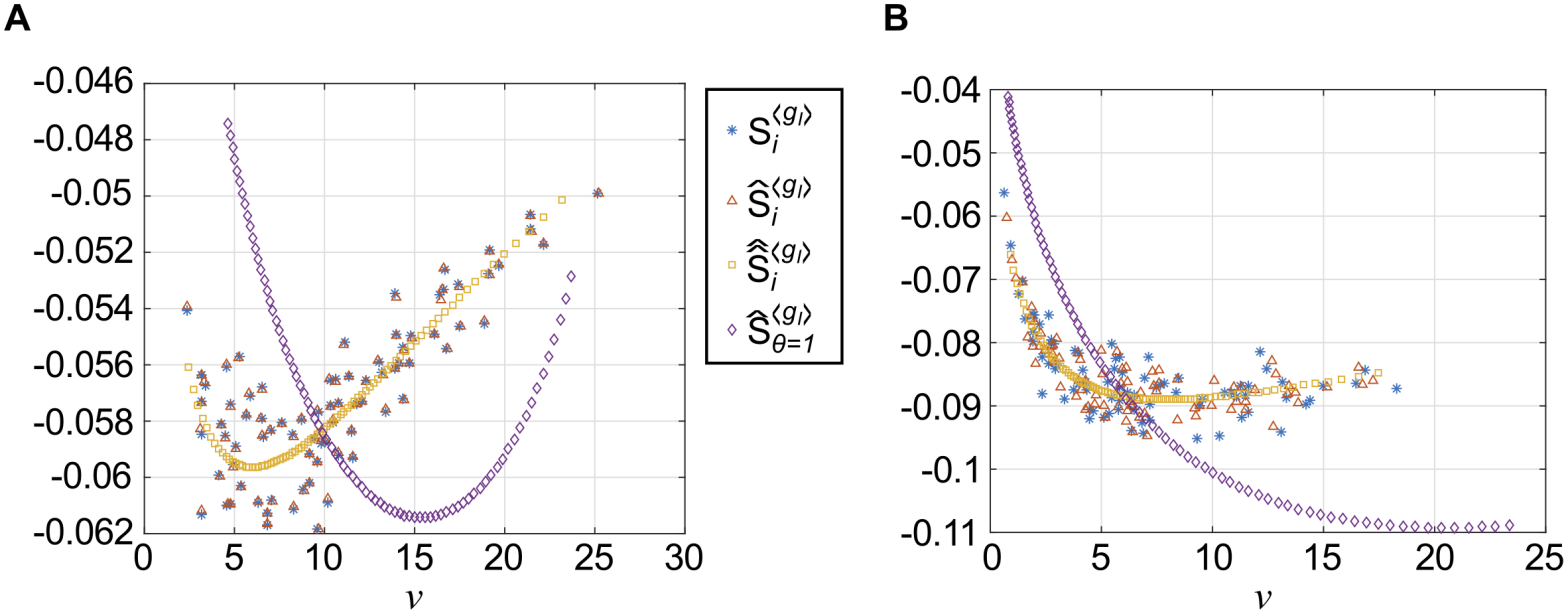
How firing rate diversity is achieved in a heterogeneous network will affect susceptibility. Single-cell susceptibility function(s) for a conductance-based LIF neuron, as a function of firing rate *ν*. Successive approximations shown are: original single-cell susceptibility, $S_{i}^{\langle g_{I}\rangle }$ (Eq 4, blue stars); most parameters set to average value, ${\hat{S}}_{i}^{\langle g_{I}\rangle }$ (Eq 7, red triangles); all parameters but *θ*_*i*_ set to average value, ${\hat{\hat{S}}}_{i}^{\langle g_{I}\rangle }$ (Eq 9, gold squares); and *θ* fixed, ${\hat{S}}_{\theta =1}^{\langle g_{I}\rangle }$ (Eq 10, purple diamonds). (A) Asynchronous regime. (B) Strong asynchronous regime.

This quantity is shown in Fig 6, where it is plotted vs. firing rate *ν*_*i*_ (blue stars). Note that this is a negative quantity; since the susceptibility for a neuron pair $S_{ij}^{\langle g_{I}\rangle }=S_{i}^{\langle g_{I}\rangle }S_{j}^{\langle g_{I}\rangle }$ is the product (and therefore positive), an *increase* in $S_{i}^{\langle g_{I}\rangle }$ will result in a *decrease* in $S_{ij}^{\langle g_{I}\rangle }$ and vice versa.

In principle, the firing rate function (Eq 5)—and therefore susceptibility—can depend on all *six* parameters defining the cell: our next step was to reduce the dimensionality of the problem. We first looked for any possible relationship between single-cell firing rates and cell parameters (see S1 Text**: Approximating single-cell susceptibility in a heterogeneous network**, S6 and S7 Figs): in both networks, only threshold *θ*_*i*_ had an obvious relationship with firing rate. Among the remaining parameters, the mean inhibitory conductance 〈*g*_*I*_〉 had the greatest relative range of values in the asynchronous network (S6A Fig). Therefore, we hypothesized that we could accurately capture $S_{i}^{\langle g_{I}\rangle }$, by approximating it as a function of the two parameters *θ*_*i*_ and 〈*g*_*I*_〉.

**Fig 7:**
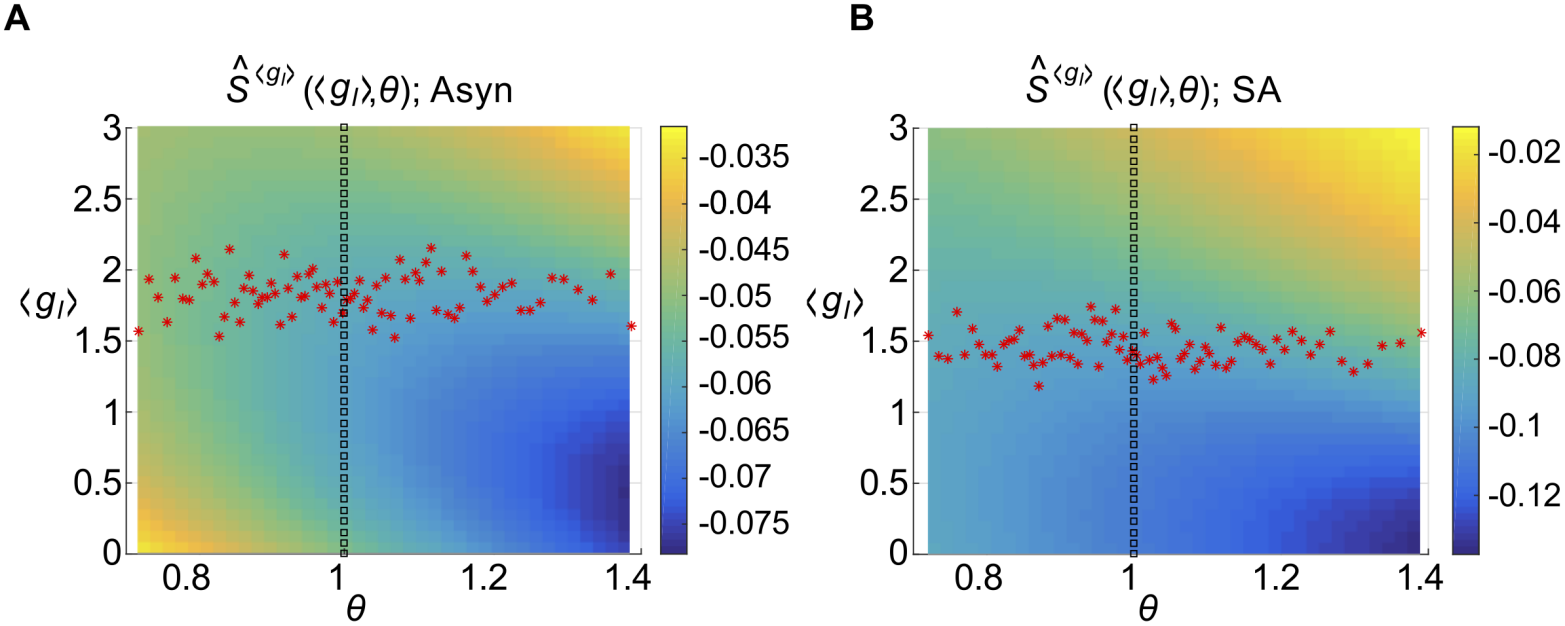
Susceptibility as a function of inhibitory conductance and threshold. Single-cell susceptibility function for a conductance-based LIF neuron, as a function of mean inhibitory conductance 〈*g*_*I*_〉 and threshold *θ*: ${\hat{S}}^{\langle g_{I}\rangle }\left(\langle g_{I}\rangle ,\theta \right)$ (defined in Eq 7). Other parameters are set to the population average. Overlays show (〈*g*_*I*,*i*_〉, *θ*_*i*_) values of the actual cells in the network (red stars) and an alternative curve through the plane, (〈*g*_*I*_〉, 1), along which comparable firing rate diversity can be observed (black squares). (A) Asynchronous regime. (B) Strong asynchronous regime.

We reevaluated the firing rate function, where *σ*_*I*,*i*_, 〈*g*_*E*,*i*_〉, *σ*_*E*,*i*_ and *σ*_*i*_ have been replaced by their average values: i.e.

$${\hat{S}}_{i}^{\left\langle g_{I}\right\rangle }\equiv \frac{1}{\sqrt{F(\left\langle g_{I,i}\right\rangle ,\theta_{i})}}\frac{\partial F}{\partial x_{1}}\left(\left\langle g_{I,i}\right\rangle ,\theta_{i}\right)$$ (7)

where

$$F\left(\left\langle g_{I,i}\right\rangle ,\theta_{i}\right)\equiv f\left(\left\langle g_{I,i}\right\rangle ,{\left\langle \sigma_{I,i}\right\rangle }_{p},{\left\langle \;\left\langle g_{E,i}\right\rangle \;\right\rangle }_{p},{\left\langle \sigma_{E,i}\right\rangle }_{p},{\left\langle \sigma_{i}\right\rangle }_{p},\theta_{i}\right)$$ (8)

and 〈 ⋅ 〉_*p*_ denotes the population average. The results are also illustrated in Fig 6 (red triangles). In the asynchronous regime (Fig 6A), the results are remarkably close to the original quantities, indicating that using average parameter values has little effect; in the strong asynchronous regime (Fig 6B) the difference is larger, but the points appear to occupy the same “cloud”. However, we can now visualize the susceptibility as a function of only *two* parameters, and we do so in Fig 7 by evaluating ${\hat{S}}_{i}^{\langle g_{I}\rangle }$ on a (*θ*, 〈*g*_*I*_〉) grid; the points corresponding to the actual excitatory cells in our network are illustrated in red. In both the asynchronous and strong asynchronous regimes, the red stars form a scattered cloud around the average value 〈〈*g*_*I*,*i*_〉〉_*p*_, with no obvious relationship with *θ*_*i*_.

This fact motivated a further simplification,

$${\hat{\hat{S}}}_{i}^{\left\langle g_{I}\right\rangle }\equiv \frac{1}{\sqrt{F({\left\langle \;\left\langle g_{I,i}\right\rangle \right\rangle }_{p},\theta_{i})}}\frac{\partial F}{\partial x_{1}}\left({\left\langle \;\left\langle g_{I,i}\right\rangle \;\right\rangle }_{p},\theta_{i}\right)$$ (9)

i.e., we replaced 〈*g*_*I*,*i*_〉 with its population average, 〈〈*g*_*I*,*i*_〉〉_*p*_, in essence approximating a one-dimensional “path” that the cells take through parameter space. The results are shown in Fig 6 (gold squares) and, as we should expect, allow us to discern a functional relationship with firing rate *ν*_*i*_; importantly, it appears to capture the average behavior of the actual susceptibility values $S_{i}^{\langle g_{I}\rangle }$. Here, we can see clearly that in the asynchronous regime, correlations should actually *decrease* with firing rate, for *ν*_*i*_ > 5 Hz. In the strong asynchronous regime, correlations will *increase* with firing rate, saturating around 10–15 Hz.

### Susceptibility depends on the mechanism underlying firing rate diversity

Finally, recall that our actual network sampled a relatively small part of the (*θ*, 〈*g*_*I*_〉) plane. This may be attributed to the fact that we generated firing rate diversity (and therefore heterogeneity), by modulating cell excitability through the cell threshold *θ*_*i*_. How might our results have changed, if we had generated firing rate diversity through some other mechanism? In both regimes, we can increase firing rates by either decreasing 〈*g*_*I*,*i*_〉, or by decreasing *θ* (see S7 Fig). To explore this, we computed susceptibility values along another curve in the (*θ*, 〈*g*_*I*_〉) plane; specifically, we held *θ* fixed and instead varied 〈*g*_*I*_〉 (illustrated with black squares on Fig 7); i.e.

$${\hat{S}}_{\theta =1}^{\left\langle g_{I}\right\rangle }\left(\left\langle g_{I}\right\rangle \right)\equiv \frac{1}{\sqrt{G(\left\langle g_{I}\right\rangle ,\theta )}}\frac{\partial G}{\partial x_{1}}\left(\langle g_{I}\rangle ,\theta \right){|}_{\theta =1}$$ (10)

where

$$\;G\left(\left\langle g_{I}\right\rangle ,\theta \right)=f\left(\left\langle g_{I}\right\rangle ,{\left\langle \sigma_{I,i}\right\rangle }_{p},{\left\langle \;\left\langle g_{E,i}\right\rangle \;\right\rangle }_{p},{\left\langle \sigma_{E,i}\right\rangle }_{p},{\left\langle \sigma_{i}\right\rangle }_{p},\theta \right)$$

Results are shown in Fig 6 (purple diamonds) and show a strikingly different relationship with firing rate; in the asynchronous regime, correlations should increase with firing rate for *ν* < 15 Hz; in the strong asynchronous regime correlations will increase with firing rate, saturating near 20 Hz.

To summarize the previous two subsections, we first defined a single-cell susceptibility function (Eq 4), which captures a linear approximation to the cell’s response to input. This quantity relies on an underlying firing rate, which is a function of all parameters that define single-cell dynamics; in this case, six. Each cell occupies a point in this six-dimensional parameter space. We found that in each network studied here, the occupied points approximately lie along a one-dimensional path through this parameter space, along which we could visualize the susceptibility. Finally, we considered the consequences of taking other paths through this parameter space: these paths can be interpreted as generating firing rate heterogeneity using other network mechanisms.

### Low-rank structure in neural correlations is mediated by correlation-firing rate relationship

Previous work has identified low-dimensional structure in neural correlation matrices [25, 38–41]; its origin is not always known [3]. We next hypothesized that the positive correlation-firing rate relationship we observed in the strong asynchronous regime, might be reflected in low-dimensional structure in the correlation matrix. For simplicity, suppose that correlations were really represented by a function of firing rate (as in [22]): i.e. *ρ*_*ij*_ = *cS*(*ν*_*i*_)*S*(*ν*_*j*_). Then we could represent the *off-diagonal* part of the correlation matrix as **C**_*T*_ = *c***SS**^*T*^, where **S** is a length *N* vector such that **S**_*i*_ = *S*(*ν*_*i*_); that is, **C**_*T*_ would be a rank-one matrix.

We followed the procedure outlined in **Methods: Low-rank approximation to the correlation matrix** to approximate each correlation matrix, **C**_*T*_, as the sum of a diagonal matrix and low-rank matrix:

$$C_{T}\;\approx \;C_{T}^{\text{diag}+R1}=\lambda I+\left(\sigma_{1}-\lambda \right)u_{1}u_{1}^{T}$$ (11)

where λ is given in closed form by the eigenvalues of **C**_*T*_:

$$\lambda =\lambda_{1}-\frac{\sum_{j>1}{\left(\lambda_{1}-\lambda_{j}\right)}^{2}}{\sum_{j>1}\lambda_{1}-\lambda_{j}}$$ (12)

and *σ*_1_, **u**_1_ are the first singular value and singular vector of **C**_*T*_.

**Fig 8:**
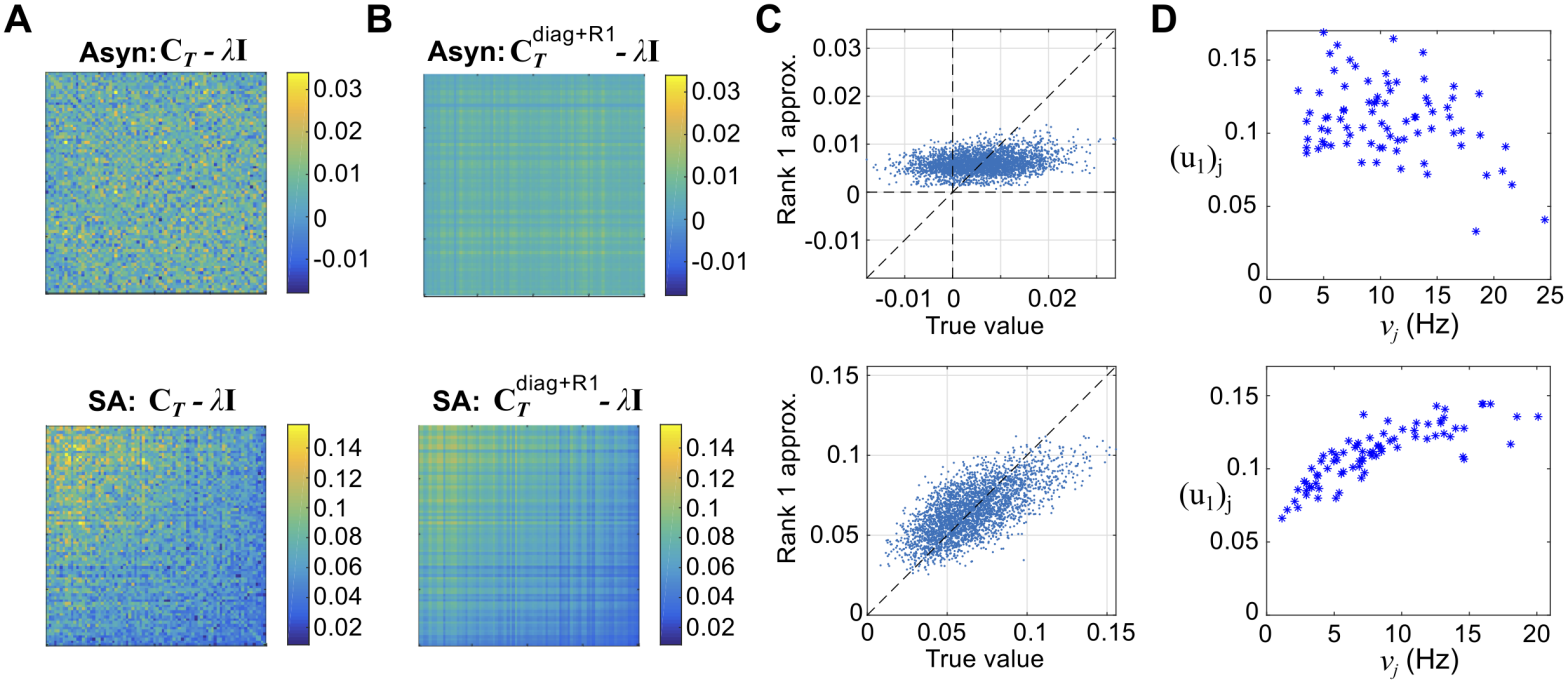
Low-rank structure in correlation matrices. Approximating correlation matrices for the heterogeneous networks as a diagonal plus rank-one. Neurons are ordered by firing rate (highest to lowest). In each column of (A-D), the asynchronous (top) and strong asynchronous (bottom) regimes are shown; *T* = 100 ms.(A) The shifted E-E correlation matrix, **C**_*T*_ − λ**I**, for an appropriately chosen λ. (B) A rank-one approximation to **C**_*T*_ − λ**I**. (C) True correlation coefficients vs. rank-one approximation, cell-by-cell. (D) Weight in the first singular vector, **u**_1_ vs. geometric mean firing rate $\sqrt{\nu_{i}\nu_{j}}$.

In Fig 8, we show the results from heterogeneous networks in both the asynchronous (top panel in each subfigure) and strong asynchronous (bottom panel in each subfigure) regimes. We first show **C**_*T*_ − λ**I**, where λ is given by Eq 12, in Fig 8A. Cells are ordered by (decreasing) firing rate. While no pattern is visible in the asynchronous state (top panel), the strong asynchronous state (bottom panel) shows larger values in the upper left corner, suggesting that correlation increases with firing rate. This is even more visible in the rank one approximation, $\left(\sigma_{1}-\lambda \right)u_{1}u_{1}^{T}$, shown in Fig 8B.

We now use $C_{T}^{\text{diag}+R1}$ to approximate **C**_*T*_, and compare the results, cell pair-by-cell pair (Fig 8C). In the asynchronous network, the approximated correlations take on a narrow range (between 0 and 0.01, compared to between −0.015 and 0.03 for the measured coefficients) and do not show an obvious positive relationship. In the strong asynchronous regime, the range is more accurate (between 0.02 and 0.1, vs. 0.01 and 0.15 for the measured coefficients) and the points cluster around the unity line.

In Fig 8D, we plot the weight of each cell in the first singular vector, (**u**_1_)_*j*_ vs. the firing rate *ν*_*j*_. We can clearly see a positive relationship in the strong asynchronous regime (bottom panel), suggesting that the positive relationship between correlation and firing rate is related to the success of the low-rank approximation.

## Discussion

We simulated heterogeneous, asynchronous networks of leaky integrate-and-fire model neurons in order to investigate a possible relationship between firing rates and pairwise correlations in recurrent networks. We found that correlations can either increase *or* decrease with firing rates; this could be attributed to differences in how cells responded to fluctuations in inhibitory conductances. When correlations *did* increase with firing rates, this relationship was reflected in low-dimensional structure in the correlation matrix.

This study offers an example of a practical consequence of the difference between treating synaptic inputs as *conductances* rather than *currents*; while most synaptic currents are more accurately modeled as conductances, current-based formulations are often used for analytical and computational simplicity. Although it is known that neural models responding to currents vs. conductances differ in their response dynamics [44–46], this approach is supported by findings that steady-state firing rates are qualitatively similar in both settings (e.g. [47]). Here, we found that refined features of the steady-state firing rate surface will govern susceptibility to common input in asynchronous networks; two “cuts” through this surface may yield divergent behavior with respect to correlation susceptibility, despite yielding similar firing rates.

In other words, the relationship between pairwise correlations and firing rate will depend on the means through which firing rate diversity is achieved. In our study, we created firing rate diversity by regulating cell excitability; if we had instead varied mean inhibitory input (by varying the number of inhibitory connections) or a background excitatory current (which would model diversity in stimulus tuning from feed-forward inputs), we would likely have seen a different pattern. Finally, the recurrent network will also shape the path the cells follow through the “firing rate surface”; to generalize [22] to recurrent networks, we need to identify both how firing rates are produced and how they are shaped by the recurrent network.

Thus far, we have not directly connected the presence or absence of a positive correlation-firing rate relationship to other firing statistics (such as being in the asynchronous vs. strong asynchronous state, for example). We believe this will be a challenging question to answer; indeed, we showed earlier (in Results**: Susceptibility depends on the mechanism underlying firing rate diversity**) that we can construct a network with asynchronous firing, but where correlations *do* increase with firing rate. Therefore, our goal with this paper is to present a detailed procedure for analyzing how correlations will vary with firing rates in recurrent networks, along with a few illustrative (and nonintuitive) examples.

Finally, while the networks considered in this paper had fixed in-degrees (rather than Erdős-Rényi), this is not necessary. We have reproduced these results in Erdős-Rényi networks, in which parameters are identical to those chosen here, except that each network connection was chosen independently with a probability that depended only on E/I identity. In S9 Fig, we show correlations vs. geometric firing rates, for all excitatory pairs (as in Fig 2), confirming that correlations increased with firing rates in the strong asynchronous network, but not in the asynchronous network. Furthermore, we hypothesize that any difference between fixed in-degree and Erdős-Rényi networks will become less rather than more important with increasing network size, as the variance in synaptic inputs decreases.

Low-dimensional structure has been a common finding in many large-scale neural recordings [25, 38–41]; while the origin is not always known, it is often interpreted as arising from a global input or top-down signal. This is an interpretation that arises naturally from the technique of *factor analysis*, in which one seeks to explain a data vector as the sum of a random vector and the linear combination of some number of latent factors [48] (for Gaussian random variables, each latent factor can literally be interpreted as a global input with a distinct pattern of projection onto the observed variables). In our network, we found that a single latent factor was effective at capturing correlations in the strong asynchronous regime; however, this latent factor did not reflect common input (there was no global external input into the network) but rather modulation from single-cell characteristics. Thus, we identify a novel mechanism that can contribute to low-dimensional structure in neural recordings.

### Impact on stimulus coding

The networks studied here were not encoding a stimulus; correlations were generated by recurrent activity, given that each neuron had a baseline firing rate in the absence of recurrent input. However, we can readily connect this network to a stimulus coding task, in order to understand how the correlation-firing rate relationship can impact coding.

Consider a population of cells that is responsible for encoding a single scalar stimulus *θ*, such as movement direction or orientation of a visual stimulus, and that each cell has roughly a bell-shaped tuning curve. Furthermore, we model an incoming stimulus by modulating a stimulus-dependent background current *I*_*i*,_(*θ*); i.e., cells which prefer the current stimulus have a higher level of current, and thus a higher firing rate, than cells which prefer an orthogonal or opposite stimulus. The network we studied here would model the response to a single stimulus *θ*_0_; that is, the firing rate diversity we observe is present because some cells are strongly tuned to the current stimulus, while others are not.

We could extend this model, by resetting background currents to model a complete set of stimuli {*θ*_1_, *θ*_2_, …*θ*_*n* − 1_}. For each stimulus *θ*_*j*_, correlations would show the rough firing rate dependence displayed in the strong asynchronous network, resulting in a *stimulus-dependent* correlation structure in which pairwise correlations vary like geometric mean firing rate. This is the structure analyzed in [21, 27]: the authors found that such a stimulus-dependent correlation code enhances information, when compared to a stimulus-independent code with the same average correlation level. Intuitively, the mean population response lives on the surface of a (hyper-)sphere in neural response space; the population encodes location on this surface. Positive correlations between similarly tuned cells produce response distributions that are stretched in the radial direction, “orthogonal” to this sphere, and thus have a minimal impact on the encoded variable.

Moreover, the mechanism that produced stimulus-dependent correlations in [21, 27] was similar to that shown here (see also [25]); common input modulated by stimulus-dependent gain factors. Here, we demonstrated how these stimulus-dependent gain factors might arise (or not) in a recurrent network. If excitation is tuned to put the network in the strong asynchronous regime, then the (stimulus-dependent) correlation structure that results will be favorable to coding. If excitation is tuned to put the network in the asynchronous regime, then correlations are overall low and not stimulus-dependent (although, given that average correlations are not matched, we do not here compare information contained within the two networks).

### Future work

This work has, necessarily, focused only on a subset of network attributes that might affect firing statistics. One important feature is the frequency of higher-order graph motifs; experiments have shown that specific motifs will occur more frequently, than would be expected in an Erdős-Rényi network with fixed single-cell connection probability [49]. Theoretical work has found that in networks of integrate-and-fire neurons, an overabundance of divergent and chain motifs will lead to enhanced correlation [33] (this finding does depend on the dynamical regime; different motifs impact correlations in networks of coupled oscillators [32]). In [33], the authors use the assumption of homogeneous single-cell characteristics to find parsimonious and instructive formulae for the average correlation, and give a roadmap for how this might be generalized to heterogeneous networks. We look forward to considering the combined effect of single-cell *and* network heterogeneity in future work.

Another source of cell-to-cell heterogeneity is how cells respond to stimuli, as emphasized in the previous discussion [17, 20, 21, 27, 50] (see [19] for a review). Here, we did not consider a specific sensory system with tuning but rather focus on the general question of how the distribution of correlation values arise in recurrent networks. Given the previous discussion, one next step will be to investigate how correlations covary with firing rates, when cell-to-cell heterogeneity is produced by stimulus tuning in a structured network responding to a single variable (such as direction or orientation).

Finally, for numerical tractability our simulations here were performed in relatively small networks. While high average correlations have been measured in experiments [51], theoretical models of asynchronous networks have found that correlations must go to zero as the system becomes large (*N* → ∞) [2]. However, recent work has found that this does not have to be true, as long as spatial structure is introduced into the network [52]. We anticipate that this may carry over to other forms of heterogeneity, such as single-cell variability, and that therefore the effect we observe here persists for larger networks. We look forward to reporting on this in future work.

## Methods

### Neuron model and network setup

We considered randomly connected networks of excitatory and inhibitory neurons. Each cell was a linear integrate-and-fire model with second-order alpha-conductances, i.e. membrane voltage *ν*_*i*_ was modeled with a stochastic differential equation, as long as it remained beneath a threshold *θ*_*i*_:

$$\tau_{m}\frac{d\nu_{i}}{dt}=-\nu_{i}-g_{E,i}\left(t\right)\left(\nu_{i}-E_{E}\right)-g_{I,i}\left(t\right)\left(\nu_{i}-E_{I}\right)+\sigma_{i}\sqrt{\tau_{m}}\xi_{i}\left(t\right),$$ (13)

When *ν*_*i*_ reaches *θ*_*i*_, it is reset to 0 following a refractory period:

$$\nu_{i}\left(t+\tau_{\text{ref}}\right)\to 0,\;\nu_{i}\left(t\right)\geq \theta_{i}$$ (14)

Each neuron was driven by a Gaussian, white background noise, with magnitude *σ*_*i*_ depending only on the cell type; that is, 〈*ξ*_*i*_(*t*)〉 = 0 and 〈*ξ*_*i*_(*t*)*ξ*_*i*_(*t* + *s*)〉 = *δ*(*s*). The membrane time constant, *τ*_*m*_, and excitatory and inhibitory synaptic reversal potentials, $E_{E}$ and $E_{I}$, are the same for every cell in the network.

Each cell responded to synaptic input through conductance terms, *g*_*E*,*i*_ and *g*_*I*,*i*_, which are each governed by a pair of differential equations:

$$\tau_{d,X}\frac{dg_{X,i}}{dt}=-g_{X,i}+g_{X,i}^{\left(1\right)}$$ (15)

$$\tau_{r,X}\frac{dg_{X,i}^{\left(1\right)}}{dt}=-g_{X,i}^{\left(1\right)}+\tau_{r,X}\alpha_{X}\left(\frac{W_{YX}}{N_{YX}}\right)\sum_{j\in X,j\to i}\sum_{k}\delta \left(t-t_{j,k}\right)$$ (16)

where *Y* = {*E*, *I*} denotes the type of cell *i* and *X* = {*E*, *I*} denotes the type of the source neuron *j*. Each spike is modeled as a delta-function that impacts the auxiliary variable $g_{X,i}^{\left(1\right)}$; here *t*_*j*,*k*_ is the *k*-th spike of cell *j*. The rise and decay time constants *τ*_*r*,*X*_ and *τ*_*d*,*X*_ and pulse amplitude *α*_*X*_ depend only on the type of the source neuron; i.e. they are otherwise the same across the population. The parameter *W*_*YX*_ denotes the strength of *X* → *Y* synaptic connections, which are (once given the type of source and target neurons) identical across the population. The “raw” synaptic weight (listed in Table 1) is divided by *N*_*YX*_, the total number of *X* → *Y* connections received by each *Y*-type cell.

We chose connections to be homogeneous and relatively dense, consistent with the local architecture of cortex. Connection probabilities ranged from 20%–40%, consistent with experimentally measured values [53–55]. For our baseline network state, we then chose synaptic weights so the network is moderately inhibition-dominated (*α*_*E*_*W*_*IE*_ < *α*_*I*_*W*_*II*_ and *α*_*E*_*W*_*EE*_ < *α*_*I*_*W*_*EI*_); that is both *E* and *I* cells receive more inhibition than excitation) and shows noisy spiking consistent with the classical asynchronous state. Each neuron receives a fixed number of incoming connections, the identities of which are chosen randomly. (The specific cell ID numbers differ in the different simulations shown below.) For most of the networks we discuss here *N* = 100 with the 80/20 ratio typical of cortex (i.e *n*_*E*_ = 80, *n*_*I*_ = 20). Each excitatory cell receives *N*_*EE*_ = 32 (40%) excitatory and *N*_*EI*_ = 7 (35%) inhibitory connections; each inhibitory cell receives *N*_*IE*_ = 16 (20%) and *N*_*II*_ = 8 (40%) inhibitory projections.

In heterogeneous networks, the threshold *θ*_*i*_ varied across the population. For both excitatory and inhibitory neurons, the thresholds *θ*_*i*_ were chosen from a log-normal distribution between 0.7 and 1.4 (where the rest potential, *V*_*r*_ = 0). To be precise, log *θ*_*i*_ was chosen from a (truncated) normal distribution with mean $-s_{\theta }^{2}/2$ and standard deviation *s*_*θ*_. With this choice, *θ*_*i*_ has mean 1 and variance: $e^{s_{\theta }^{2}}-1$. Thus we can view *s*_*θ*_ as a measure of the level of threshold heterogeneity.

Throughout this paper, we set *s*_*θ*_ = 0.2, which results in a wide range of firing rates compared to the homogeneous case. This was the only source of cell-to-cell heterogeneity; all other parameters were identical across the population, conditioned on neuron type (values listed in Table 2). In homogeneous networks, the threshold was the same across the population: *θ*_*i*_ = 1.

**Table 2:** Other parameters used in network simulations.

Parameter	Definition	*X* = *E*	*X* = *I*
*τ*_*r*,*X*_	Synaptic rise time	1 ms	2 ms
*τ*_*d*,*X*_	Synaptic decay time	5 ms	10 ms
*τ*_*m*_	Membrane time constant	20 ms	20 ms
*τ*_*ref*_	Refractory time	2 ms	2 ms
*α*_*X*_	Pulse amplitude	1	2
$E_{X}$	Synaptic reversal potential	6.5	-0.5

Monte Carlo simulations were performed using the stochastic forward- Euler method (Euler-Maruyama), with a time step much smaller than any time scale in the system (Δ*t* = 0.01 ms). Each network was simulated for one second of simulation time, after an equilibration time. Then, a large number of realizations of this interval (*n*_*R*_ = 10^5^) were simulated. Spike counts were retained in each 1 ms window (for a total of 1000 windows) within a realization. With this large number of realizations/trials, the error bars on the resulting time-dependent firing rates were small. Therefore we emphasize that the firing rate pattern is largely driven by network connectivity; while firing is driven by random fluctuations in the background noise, any cell-to-cell variability in the *trial-averaged* firing rates are not an artifact of the finite number of trials.

### Linear response theory

In general, computing the response of even a single neuron to an input requires solving a complicated, nonlinear stochastic process. However, it often happens that the presence of background noise linearizes the response of the neuron, so that we can describe this response as a perturbation from a background state. This response is furthermore linear in the perturbing input and thus referred to as *linear response* theory [56]. The approach can be generalized to yield the dominant terms in the coupled network response, as well; we will use the theory to predict the covariance matrix of activity.

We first consider the case of a single cell: an LIF neuron responding to a mean zero current *ϵX*_*i*_(*t*)

$$\tau_{m}\frac{d\nu_{i}}{dt}=-\left(\nu_{i}-E_{L}\right)+E_{i}+\sigma_{i}\sqrt{\tau_{m}}\xi_{i}\left(t\right)+\epsilon X_{i}\left(t\right).$$

(otherwise, the mean of *X*_*i*_ can simply be absorbed into *E*_*i*_).

For a fixed input *ϵX*_*i*_(*t*), the output spike train *y*_*i*_(*t*) will be slightly different for each realization of the noise *ξ*_*i*_(*t*) and initial condition *ν*_*i*_(0). Therefore we try to work with the time-dependent firing *rate*, *ν*_*i*_(*t*) ≡ 〈*y*_*i*_(*t*)〉, which is obtained by averaging over all realizations and initial conditions. Linear response theory proposes the ansatz that the firing rate can be described as a perturbation from a baseline rate proportional to the input *ϵX*_*i*_:

$$\nu_{i}\left(t\right)=\nu_{i,0}+\left(A_{i}*\epsilon X_{i}\right)\left(t\right);$$ (17)

*ν*_*i*,0_ is the baseline rate (when *X* = 0) and *A*_*i*_(*t*) is a *susceptibility function* that characterizes this firing rate response up to order *ϵ* [22, 29, 57].

We now consider the theory for networks; here cell *i* responds to the spike train of cell *j*, *y*_*j*_(*t*), via the synaptic weight matrix **W**, after convolution with a synaptic filter *F*_*j*_(*t*):

$$\tau_{m}\frac{d\nu_{i}}{dt}=-\left(\nu_{i}-E_{L}\right)+E_{i}+\sigma_{i}\sqrt{\tau_{m}}\xi_{i}\left(t\right)+\sum_{j}W_{ij}F_{j}*y_{j}\left(t\right)$$

In order to consider joint statistics, we need the trial-by-trial response of the cell. We first propose to approximate the response of each neuron as:

$$y_{i}\left(t\right)\approx y_{i}^{0}\left(t\right)+\left(A_{i}*\sum_{j}\left(J_{ij}*y_{j}\right)\right)\left(t\right);$$ (18)

that is, each input *X*_*i*_ has been replaced by the synaptic input, and **J**_*ij*_ = **W**_*ij*_*F*_*j*_(*t*) includes both the *i* ← *j* synaptic weight *W*_*ij*_ and synaptic kernel *F*_*j*_ (normalized to have area 1); *A*_*i*_(*t*) is the susceptibility function from Eq 17. In the frequency domain this becomes

$${\tilde{y}}_{i}\left(\omega \right)={\tilde{y}}_{i}^{0}+{\tilde{A}}_{i}\left(\omega \right)\left(\sum_{j}{\tilde{J}}_{ij}\left(\omega \right){\tilde{y}}_{j}\left(\omega \right)\right)$$ (19)

where ${\tilde{y}}_{i}=F[y_{i}-\nu_{i}]$ is the Fourier transform of the mean-shifted process (*ν*_*i*_ is the average firing rate of cell *i*) and $\tilde{f}=F[f]$ for all other quantities. In matrix form, this yields a self-consistent equation for $\tilde{y}$ in terms of ${\tilde{y}}^{0}$:

$$\left(I-\tilde{K}\left(\omega \right)\right)\tilde{y}={\tilde{y}}^{0}\Rightarrow \tilde{y}={\left(I-\tilde{K}\left(\omega \right)\right)}^{-1}{\tilde{y}}^{0}$$ (20)

where ${\tilde{K}}_{ij}\left(\omega \right)={\tilde{A}}_{i}\left(\omega \right){\tilde{J}}_{ij}\left(\omega \right)$ is the interaction matrix, in the frequency domain. The cross-spectrum is then computed

$$\left\langle \tilde{y}\left(\omega \right){\tilde{y}}^{*}\left(\omega \right)\right\rangle ={\left(I-\tilde{K}\left(\omega \right)\right)}^{-1}\left\langle {\tilde{y}}^{0}\left(\omega \right){\tilde{y}}^{0*}\left(\omega \right)\right\rangle {\left(I-{\tilde{K}}^{*}\left(\omega \right)\right)}^{-1}$$ (21)

To implement this calculation, we first solve for a self-consistent set of firing rates: that is, *ν*_*i*_ is the average firing rate of

$$\tau_{m}\frac{d\nu_{i}}{dt}=-\left(\nu_{i}-E_{L}\right)+\left(E_{i}+E\left[f_{i}\right]\right)+\sigma_{i}\sqrt{\tau_{m}}\xi_{i}\left(t\right)$$ (22)

where E[*f*_*i*_] = ∑_*j*_ **W**_*ij*_*ν*_*j*_.

We must then compute the power spectrum $\langle {\tilde{y}}^{0}\left(\omega \right){\tilde{y}}^{0*}\left(\omega \right)\rangle$ and the susceptibility *A*_*i*_(*ω*), which is the (first order in *ϵ*) response in the firing rate $r_{i}\left(t\right)=r_{i}^{0}+\epsilon A_{i}\left(\omega \right)\exp\left(ı\omega t\right)$ in response to an input current perturbation *X*(*t*) = *ϵ* exp(*ıωt*) (here *ı* is used for $\sqrt{-1}$, while *i* denotes an index). Both can be expressed as the solution to (different) first-order boundary value problems and solved via Richardson’s threshold integration method [47, 58].

In our simulations, we used conductance-based neurons; this requires modification, compared with the simpler current-based models. We first approximate each conductance-based neuron as an effective current-based neuron with reduced time constant, following the discussion in [59]. First, separate each conductance into mean and fluctuating parts; e.g. *g*_*E*,*i*_ → 〈*g*_*E*,*i*_〉 + (*g*_*E*,*i*_ − 〈*g*_*E*,*i*_〉). Then we identify an effective conductance *g*_0,*i*_ and potential *μ*_*i*_, and treat the fluctuating part of the conductances as noise, i.e. *g*_*E*,*i*_ − 〈*g*_*E*,*i*_〉→*σ*_*E*,*i*_ *ξ*_*E*,*i*_(*t*):

$$\tau_{m}\frac{d\nu_{i}}{dt}=-g_{0,i}\left(\nu_{i}-\mu_{i}\right)+\sigma_{E,i}\xi_{E,i}\left(t\right)\left(\nu_{i}-E_{E}\right)+\sigma_{I,i}\xi_{I,i}\left(t\right)\left(\nu_{i}-E_{I}\right)+\sqrt{\sigma_{i}^{2}\tau_{m}}\xi_{i}\left(t\right)$$ (23)

where

$$g_{0,i}=1+\left\langle g_{E,i}\right\rangle +\left\langle g_{I,i}\right\rangle$$ (24)

$$\mu_{i}=\frac{E_{L}+E_{i}+\left\langle g_{E,i}\right\rangle E_{E}+\left\langle g_{I,i}\right\rangle E_{I}}{g_{0,i}}$$ (25)

$$\sigma_{E,i}^{2}=\mathrm{Var}\left[g_{E,i}\left(t\right)\right]=E\left[{\left(g_{E,i}\left(t\right)-\left\langle g_{E,i}\right\rangle \right)}^{2}\right]$$ (26)

$$\sigma_{I,i}^{2}=\mathrm{Var}\left[g_{I,i}\left(t\right)\right]=E\left[{\left(g_{I,i}\left(t\right)-\left\langle g_{I,i}\right\rangle \right)}^{2}\right]$$ (27)

We next simplify the noise terms by writing

$$\nu_{i}-E_{E}=\nu_{i}-\mu_{i}+\mu_{i}-E_{E}$$ (28)

and assume that the fluctuating part of the voltage, *ν*_*i*_ − *μ*_*i*_, is mean-zero and uncorrelated with the noise terms *ξ*_*E*,*i*_(*t*) [59]. That allows us to define an effective equation

$$\tau_{\text{eff},i}\frac{d\nu_{i}}{dt}=-\left(\nu_{i}-\mu_{i}\right)+\sqrt{\sigma_{\text{eff},i}^{2}\tau_{\text{eff},i}}\eta_{\text{eff},i}\left(t\right)$$ (29)

where

$$\tau_{\text{eff},i}=\frac{\tau_{m}}{g_{0,i}}$$ (30)

$$\sigma_{\text{eff},i}^{2}=\frac{\sigma_{E,i}^{2}{\left(\mu_{i}-E_{E}\right)}^{2}+\sigma_{I,i}^{2}{\left(\mu_{i}-E_{I}\right)}^{2}+\sigma_{i}^{2}\tau_{m}}{g_{0,i}\tau_{m}}$$ (31)

and the fluctuating voltage, *ν*_*i*_(*t*) − *μ*_*i*_, now makes no contribution to the effective noise variance.

Finally, we consider how to model the conductance mean and variance, e.g. 〈*g*_*E*,*i*_〉 and $\sigma_{E,i}^{2}$. In our simulations, we used second order *α*-functions: each conductance *g*_*X*,*i*_ is modeled by two equations that take the form

$$\tau_{r,X}\frac{dg_{X,i}^{\left(1\right)}}{dt}=-g_{X,i}^{\left(1\right)}+\tau_{r,X}{\hat{\alpha }}_{X,i}\sum_{k}\delta \left(t-t_{k}\right)$$ (32)

$$\tau_{d,X}\frac{dg_{X,i}}{dt}=-g_{X,i}+g_{X,i}^{\left(1\right)}$$ (33)

where *X* = *E*, *I* and the summation is over all type-*X* spikes incoming to cell *i*. (For notation purposes, ${\hat{\alpha }}_{X,i}$ includes all factors that contribute to the pulse size in Eq 16, including synapse strength and pulse amplitude.) The time constants *τ*_*r*,*X*_, *τ*_*d*,*X*_ may depend on synapse type; the spike jumps ${\hat{\alpha }}_{X,i}$ may depend on synapse type and target cell identity. We assume that each spike train is Poisson, with a constant firing rate: i.e. each spike train is modeled as a stochastic process *S*(*t*) with

$$\begin{array}{ccc} \left\langle S(t)\right\rangle & = & \nu \\ \left\langle S\left(t\right)S\left(t+\tau \right)\right\rangle -\nu^{2} & = & \nu \delta (\tau ) \end{array}$$

Then a straightforward but lengthy calculation shows that

$$\left\langle g_{X,i}\left(t\right)\right\rangle ={\hat{\alpha }}_{X,i}\nu_{X,i}\tau_{r,X}$$ (34)

$$\mathrm{Var}\left[g_{X,i}\left(t\right)\right]=\left(\frac{1}{2}{\hat{\alpha }}_{X,i}^{2}\nu_{X,i}\tau_{r,X}\right)\left(\frac{\tau_{r,X}}{\tau_{r,X}+\tau_{d,X}}\right)$$ (35)

where *ν*_*X*,*i*_ is the total rate of type-*X* spikes incoming to cell *i*.

We now describe how these considerations modify the linear response calculation. First, for the self-consistent firing rate calculation, Eq 22 is replaced by an equation with a modified time constant, conductance, and noise (Eq 29).

We next compute the susceptibility in response to parameters associated with the conductance, i.e. 〈*g*_*E*,*i*_〉 and $\sigma_{E,i}^{2}$. This differs from the current-based case in two ways: first, there is voltage-dependence in the diffusion terms, which results in a different Fokker-Planck equation (and thus a different boundary value problem to be solved for the power spectrum $\langle {\tilde{y}}^{0}\left(\omega \right){\tilde{y}}^{0*}\left(\omega \right)\rangle$). Second, modulating the rate of an incoming spike train will impact *both* the mean and variance of the input to the effective equation, Eq 23 (via *μ*_*i*_ and *σ*_*X*,*i*_). Furthermore, this impact may differ for excitatory and inhibitory neurons, giving us a total of *four* parameters that can be varied in the effective equation. However, neither consideration presents any essential difficulty [47].

Therefore we apply Richardson’s threshold integration method directly to Eq 23:

$$\tau_{m}\frac{d\nu_{i}}{dt}=-g_{0,i}\left(\nu_{i}-\mu_{i}\right)+\sigma_{E,i}\xi_{E,i}\left(t\right)\left(\nu_{i}-E_{E}\right)+\sigma_{I,i}\xi_{I,i}\left(t\right)\left(\nu_{i}-E_{I}\right)+\sqrt{\sigma_{i}^{2}\tau_{m}}\xi_{i}\left(t\right)$$ (36)

When we compute susceptibilities, the parameter to be varied is either a mean conductance—〈*g_E,i_*〉 → 〈*g_E,i_*〉_0_ + 〈*g_E,i_*〉_1_ exp(*ıωt*) or 〈*g_I,i_*〉 → 〈*g_I,i_*〉_0_ + 〈*g_I,i_*〉_1_ exp(*ıωt*)—or a variance—$\sigma_{E,i}^{2}\to {\left(\sigma_{E,i}^{2}\right)}_{0}+{\left(\sigma_{E,i}^{2}\right)}_{1}\exp\left(ı\omega t\right)$ or $\sigma_{I,i}^{2}\to {\left(\sigma_{I,i}^{2}\right)}_{0}+{\left(\sigma_{I,i}^{2}\right)}_{1}\exp\left(ı\omega t\right)$. Thus we have a total of four susceptibility functions ${\tilde{A}}_{\langle g_{E}\rangle ,i}\left(\omega \right)$, ${\tilde{A}}_{\langle g_{I}\rangle ,i}\left(\omega \right)$, ${\tilde{A}}_{\sigma_{E}^{2},i}\left(\omega \right)$, and ${\tilde{A}}_{\sigma_{I}^{2},i}\left(\omega \right)$. Since the Fokker-Planck equation to be solved is linear, we can compute both susceptibilities separately and then add their effects. We now have the interaction matrix:

$$\begin{array}{ccc} {\tilde{K}}_{ij}\left(\omega \right) & = & \left\{\begin{array}{cc} {\tilde{A}}_{\langle g_{E}\rangle ,i}\left(\omega \right){\tilde{J}}_{ij}\left(\omega \right)+{\tilde{A}}_{\sigma_{E}^{2},i}\left(\omega \right){\tilde{L}}_{ij}\left(\omega \right), & \;j\;\text{excitatory}\\ {\tilde{A}}_{\langle g_{I}\rangle ,i}\left(\omega \right){\tilde{J}}_{ij}\left(\omega \right)+{\tilde{A}}_{\sigma_{I}^{2},i}\left(\omega \right){\tilde{L}}_{ij}\left(\omega \right), & \;j\;\text{inhibitory} \end{array}\right. \end{array}$$ (37)

where $\tilde{L}\left(\omega \right)$ plays a similar role as $\tilde{J}$, but for the effect of incoming spikes on the *variance* of conductance. Its relationship to $\tilde{J}$ (either in the frequency or time domain) is given by the same simple scaling shown in Eq 35: i.e., for *j* excitatory,

$$\begin{array}{ccc} {\tilde{L}}_{ij}\left(\omega \right) & = & {\tilde{J}}_{ij}\left(\omega \right)\times \left(\frac{{\hat{\alpha }}_{E,i}}{2}\right)\times \left(\frac{\tau_{r,E}}{\tau_{r,E}+\tau_{d,E}}\right) \end{array}$$ (38)

where the first factor comes from the effective spike amplitude ${\hat{\alpha }}_{E,i}$ (and is the scale factor proposed in [47], Eq (64)), and the second arises from using second-order (vs. first-order) alpha-functions.

We use a modified version of the implementation given by [29] for Richardson’s threshold integration algorithm [47, 58] to compute rate *ν*_*i*_, power $\langle {\tilde{y}}_{i}^{0}\left(\omega \right){\tilde{y}}_{i}^{0*}\left(\omega \right)\rangle$, and the various susceptibilities (${\tilde{A}}_{\langle g_{E}\rangle ,i}\left(\omega \right)$, ${\tilde{A}}_{\langle g_{I}\rangle ,i}\left(\omega \right)$, ${\tilde{A}}_{\sigma_{E}^{2},i}\left(\omega \right)$, and ${\tilde{A}}_{\sigma_{I}^{2},i}\left(\omega \right)$) for an LIF neuron. We validated our code using exact formulas known for the LIF [60], and qualitative results from the literature [61].

### Computing statistics from linear response theory

Linear response theory yields the cross spectrum of the spike train, $\langle {\tilde{y}}_{i}\left(\omega \right){\tilde{y}}_{j}^{*}\left(\omega \right)\rangle$, for each distinct pair of neurons *i* and *j* (see Eq 21). To recover a representative set of statistics, we rely on several standard formulae relating this function to other statistical quantities.

The cross correlation function, **C**_*ij*_(*τ*), measures the similarity between two processes at time lag *τ*, while the cross spectrum measures the similarity between two processes at frequency *ω*:

$$C_{ij}\left(\tau \right)\equiv \left\langle \left(y_{i}\left(t\right)-\nu_{i}\right)\left(y_{j}\left(t+\tau \right)-\nu_{j}\right)\right\rangle$$ (39)

$${\tilde{C}}_{ij}\left(\omega \right)\equiv \left\langle {\tilde{y}}_{i}\left(\omega \right){\tilde{y}}_{j}\left(\omega \right)\right\rangle$$ (40)

The Weiner-Khinchin theorem [56] implies that $\left\{C_{ij},{\tilde{C}}_{ij}\right\}$ are a Fourier transform pair: that is,

$${\tilde{C}}_{ij}\left(\omega \right)=\int_{-\infty }^{\infty }C_{ij}\left(t\right)e^{-2\pi ı\omega t}\;dt$$ (41)

In principle, the crosscorrelation **C**(*t*) and cross-spectrum $\tilde{C}\left(\omega \right)$ matrices are functions on the real line, reflecting the fact that correlation can be measured at different time scales. In particular, for a stationary point process the covariance of spike counts over a window of length *T*, *n*_*i*_ and *n*_*j*_, can be related to the crosscorrelation function **C**_*ij*_ by the following formula [4]:

$${\mathrm{Cov}}_{T}\left(n_{i},n_{j}\right)=\int_{-T}^{T}C_{ij}\left(\tau \right)\left(T-|\tau |\right)\;d\tau$$ (42)

The variance of spike counts over a time window of length *T*, *n*_*i*_, is likewise given by integrating the autocorrelation function **C**_*ii*_:

$${\mathrm{Var}}_{T}\left(n_{i}\right)=\int_{-T}^{T}C_{ii}\left(\tau \right)\left(T-|\tau |\right)\;d\tau$$ (43)

It can be helpful to normalize by the time window, i.e.

$$\begin{array}{ccc} \frac{{\mathrm{Cov}}_{T}\left(n_{i},n_{j}\right)}{T} & = & \int_{-T}^{T}C_{ij}\left(\tau \right)\left(1-\frac{|\tau |}{T}\right)\;d\tau ; \end{array}$$ (44)

we can now see that for an integrable cross correlation function (and bearing in mind that the cross-spectrum is the Fourier transform of the cross correlation), that

$$\begin{array}{ccc} \lim_{T\to \infty }\frac{{\mathrm{Cov}}_{T}\left(n_{i},n_{j}\right)}{T} & = & \int_{-\infty }^{\infty }C_{ij}\left(\tau \right)d\tau \;=\;{\tilde{C}}_{ij}\left(0\right) \end{array}$$ (45)

while

$$\begin{array}{ccc} \lim_{T\to 0}\frac{{\mathrm{Cov}}_{T}\left(n_{i},n_{j}\right)}{T^{2}} & = & \frac{1}{T}\int_{-T}^{T}C_{ij}\left(\tau \right)\left(1-\frac{|\tau |}{T}\right)\;d\tau \;\approx \;C_{ij}\left(0\right) \end{array}$$ (46)

Thus, we can use ${\tilde{C}}_{ij}\left(0\right)$ and **C**_*ij*_(0) as measures of long and short time correlations respectively.

Finally, the Pearson’s correlation coefficient of the spike count defined as:

$$\begin{array}{ccc} \rho_{T,ij} & = & \frac{{\mathrm{Cov}}_{T}\left(n_{i},n_{j}\right)}{\sqrt{{\mathrm{Var}}_{T}\left(n_{i}\right){\mathrm{Var}}_{T}\left(n_{j}\right)}} \end{array}$$ (47)

is a common normalized measure of noise correlation, with *ρ* ∈ [−1, 1]. While Cov_*T*_ and Var_*T*_ grow linearly with *T* (for a Poisson process, for example), *ρ*_*T*,*ij*_ in general will not (although it may increase with *T*). In general, *ρ*_*T*,*ij*_ depends on the time window *T*; however for readability we will often suppress the *T*-dependence in the notation (and use *ρ*_*ij*_ instead).

### Quantifying the role of motifs in networks

We next explain how we can use the results of linear response theory to give insight into the role of different paths in the network. We begin with our predicted cross-spectrum (Eqs 21 and 40) and apply a standard series expansion for the matrix inverse:

$$\tilde{C}\left(\omega \right)={\left(I-\tilde{K}\left(\omega \right)\right)}^{-1}{\tilde{C}}^{0}\left(\omega \right){\left(I-{\tilde{K}}^{*}\left(\omega \right)\right)}^{-1}$$ (48)

$$=\left[\sum_{k=0}^{\infty }{\left(\tilde{K}\left(\omega \right)\right)}^{k}\right]{\tilde{C}}^{0}\left(\omega \right)\left[\sum_{l=0}^{\infty }{\left(\tilde{K}\left(\omega \right)\right)}^{l}\right]$$ (49)

$$=\sum_{k=0}^{\infty }\sum_{l=0}^{\infty }{\left(\tilde{K}\left(\omega \right)\right)}^{k}{\tilde{C}}^{0}\left(\omega \right){\left(\tilde{K}\left(\omega \right)\right)}^{l}$$ (50)

where ${\tilde{C}}^{0}\left(\omega \right)$ is a diagonal matrix containing the power spectra of the unperturbed processes; i.e. ${\tilde{C}}_{ii}^{0}\equiv \langle {\tilde{y}}_{i}\left(\omega \right){\tilde{y}}_{i}\left(\omega \right)\rangle$. This double sum will converge as long as the spectral radius of $\tilde{K}$ is less than 1 [29].

By truncating this double sum to contain terms such that *k* + *l* ≤ *n*, we define the *n*th approximation to the cross-spectrum:

$$\tilde{C}\left(\omega \right)\approx {\tilde{C}}^{n}\left(\omega \right)$$ (51)

$$={\tilde{C}}^{0}\left(\omega \right)+\sum_{k=1}^{n}\left[\sum_{l=0}^{k}{\left(\tilde{K}\left(\omega \right)\right)}^{k-l}{\tilde{C}}^{0}\left(\omega \right){\left({\tilde{K}}^{*}\left(\omega \right)\right)}^{l}\right]$$ (52)

Each distinct term in the inner sum can be attributed to a particular undirected path of length *k*. Terms of the form ${\tilde{K}}^{k}{\tilde{C}}^{0}$ and ${\tilde{C}}^{0}{\left({\tilde{K}}^{*}\right)}^{k}$ account for unidirectional paths from *j* → *i* and *i* → *j* respectively; the term ${\left(\tilde{K}\left(\omega \right)\right)}^{k-l}{\tilde{C}}^{0}\left(\omega \right){\left({\tilde{K}}^{*}\left(\omega \right)\right)}^{l}$ captures the contribution from a cell that has a length *l* path onto cell *j* and a length *k* − *l* path onto cell *i*. Thus, we can use Eq 52 to decompose the correlation into contributions from different motifs ([28], see also [31, 62]).

We can also consider the contribution from all length-*n* paths; that is,

$${\tilde{P}}^{n}={\tilde{C}}^{n}\left(\omega \right)-{\tilde{C}}^{n-1}\left(\omega \right)=\sum_{l=0}^{n}{\left(\tilde{K}\left(\omega \right)\right)}^{n-l}{\tilde{C}}^{0}\left(\omega \right){\left({\tilde{K}}^{*}\left(\omega \right)\right)}^{l}$$

If the sum in Eq 50 converges, we should expect the magnitude of contributions to decrease as *n* increases.

We will also show the *normalized* contribution from length-*n* paths, which we define as follows: let **Λ**(*ω*) be the diagonal matrix with $\Lambda_{ii}\left(\omega \right)={\tilde{C}}_{ii}\left(\omega \right)$. Then we define the matrix of contributions from length-*n* paths ${\tilde{R}}^{n}$ as follows:

$${\tilde{R}}^{n}\left(\omega \right)=\Lambda^{-1/2}\left(\omega \right){\tilde{P}}^{n}\left(\omega \right)\Lambda^{-1/2}\left(\omega \right)$$ (53)

Equivalently, ${\tilde{R}}_{ij}^{n}\left(\omega \right)={\tilde{P}}_{ij}^{n}\left(\omega \right)/\sqrt{{\tilde{C}}_{ii}\left(\omega \right){\tilde{C}}_{jj}\left(\omega \right)}$. This effectively normalizes the cross correlation by the autocorrelation; in particular, we can use this to decompose the correlation coefficient (Eq 47) for long time windows, because $\lim_{n\to \infty }\sum_{k=0}^{n}{\tilde{R}}^{k}\left(0\right)=\lim_{T\to \infty \;}\rho_{T,ij}$.

In general, we will show long-timescale correlation (e.g. $\tilde{C}\left(0\right)$ or ${\tilde{R}}^{n}\left(0\right)$) (Eq 45); results were qualitatively similar for other timescales.

### Quantifying correlation susceptibility

We next consider how to quantify the (linear) susceptibility of correlation to a change in parameter. Returning to Eq 17, but written in terms of the single-cell response:

$$y_{i}\left(t\right)=y_{i,0}+\left(A_{\mu ,i}*X_{\mu }\right)\left(t\right)\;\Rightarrow$$ (54)

$${\tilde{y}}_{i}\left(\omega \right)={\tilde{y}}_{i,0}\left(\omega \right)+{\tilde{A}}_{\mu ,i}\left(\omega \right){\tilde{X}}_{\mu }\left(\omega \right)$$ (55)

Here, *X*_*μ*_(*t*) is a (possibly) time-dependent change in a parameter, such as input current or mean inhibitory conductance; *y*_*i*,0_ is the baseline spike train (when *X* = 0). *A*_*μ*,*i*_(*t*) is a *susceptibility function* that characterizes the cell’s response (to the parameter variation) as long as *X*_*μ*_(*t*) is small [22, 29, 57]. Following [22], the cross-spectrum of *y* can now be approximated as:

$${\tilde{C}}_{ij}\left(\omega \right)\equiv \left\langle {\tilde{y}}_{i}^{*}{\tilde{y}}_{j}\right\rangle \approx \left\langle {\tilde{y}}_{i,0}^{*}{\tilde{y}}_{j,0}\right\rangle +\left\langle {\tilde{A}}_{\mu ,i}^{*}{\tilde{X}}_{\mu }^{*}{\tilde{y}}_{j,0}\right\rangle +\left\langle {\tilde{A}}_{\mu ,j}{\tilde{X}}_{\mu }{\tilde{y}}_{i,0}^{*}\right\rangle +{\tilde{A}}_{\mu ,i}^{*}{\tilde{A}}_{\mu ,j}\left\langle {\tilde{X}}_{\mu }^{*}{\tilde{X}}_{\mu }\right\rangle$$ (56)

$$={\tilde{A}}_{\mu ,i}^{*}\left(\omega \right){\tilde{A}}_{\mu ,j}\left(\omega \right){\tilde{C}}_{\mu }\left(\omega \right)$$ (57)

where ${\tilde{C}}_{\mu }\left(\omega \right)$ is the spectrum of the parameter variation. The susceptibility has an appealing interpretation in the limit *ω* → 0, as the derivative of the classical *f-I curve*:

$$\lim_{\omega \to 0}{\tilde{A}}_{\mu ,i}\left(\omega \right)=\frac{d\nu_{i}}{d\mu }$$ (58)

where *ν*_*i*_ is the steady-state firing rate of cell *i*, assuming we can measure it for specific values of the parameter *μ*.

$$\begin{array}{ccc} \lim_{T\to \infty }\rho_{T,ij} & = & \lim_{T\to \infty }\frac{{\mathrm{Cov}}_{T}\left(n_{i},n_{j}\right)}{\sqrt{{\mathrm{Var}}_{T}\left(n_{i}\right){\mathrm{Var}}_{T}\left(n_{j}\right)}}=\frac{{\tilde{C}}_{ij}\left(0\right)}{\sqrt{{\tilde{C}}_{ii}\left(0\right){\tilde{C}}_{jj}\left(0\right)}} \end{array}$$ (59)

$$\begin{array}{ccc} & \approx & \frac{{\tilde{A}}_{\mu ,i}\left(0\right){\tilde{A}}_{\mu ,j}\left(0\right)}{\sqrt{{\tilde{C}}_{ii}\left(0\right){\tilde{C}}_{jj}\left(0\right)}}{\tilde{C}}_{\mu }\left(0\right) \end{array}$$ (60)

This motivates the definition of a *correlation susceptibility*, which approximates the change in pairwise correlation induced by a parameter change experienced by both cells *i* and *j*:

$$\begin{array}{ccc} S_{ij}^{\mu } & = & \frac{{\tilde{A}}_{\mu ,i}\left(0\right){\tilde{A}}_{\mu ,j}\left(0\right)}{\sqrt{{\tilde{C}}_{ii}\left(0\right){\tilde{C}}_{jj}\left(0\right)}} \end{array}$$ (61)

If this increases with firing rate—that is, if $\frac{dS_{ij}^{\mu }}{d\nu }>0$—then correlations will also increase with firing rate.

We can further analyze this quantity by making an assumption for asynchronous spiking, that spike count variance is equal to spike count mean; i.e. ${\mathrm{Var}}_{T}\left(n_{i}\right)=T\nu_{i}\Rightarrow {\tilde{C}}_{ii}=\nu_{i}$. Then

$$\begin{array}{ccc} S_{ij}^{\mu } & \approx & \frac{1}{\sqrt{\nu_{i}\nu_{j}}}{\tilde{A}}_{\mu ,i}\left(0\right){\tilde{A}}_{\mu ,j}\left(0\right)=\frac{{\tilde{A}}_{\mu ,i}\left(0\right)}{\sqrt{\nu_{i}}}\frac{{\tilde{A}}_{\mu ,j}\left(0\right)}{\sqrt{\nu_{j}}} \end{array}$$ (62)

which motivates the definition of the single-cell quantity

$$\begin{array}{c} S_{i}^{\left\langle g_{I}\right\rangle }\equiv \frac{{\tilde{A}}_{\langle g_{I}\rangle ,i}\left(0\right)}{\sqrt{\nu_{i}}} \end{array}$$

In general, the firing rate depends on *all* single cell parameters included in Eqn.; i.e. there exists some function *f* such that

$$\nu_{i}=f\left(\left\langle g_{I,i}\right\rangle ,\sigma_{I,i},\left\langle g_{E,i}\right\rangle ,\sigma_{E,i},\sigma_{i},\theta_{i}\right)$$ (63)

$$\begin{array}{ccc} {\tilde{A}}_{\langle g_{I}\rangle ,i}\left(0\right) & = & \frac{\partial f}{\partial x_{1}}\left(\left\langle g_{I,i}\right\rangle ,\sigma_{I,i},\left\langle g_{E,i}\right\rangle ,\sigma_{E,i},\sigma_{i},\theta_{i}\right) \end{array}$$ (64)

(recall that the susceptibility for *ω* = 0 is the derivative of the firing rate with respect to the appropriate parameter (here, mean inhibitory conductance 〈*g*_*I*_〉).

### Low-rank approximation to the correlation matrix

We consider the correlation matrix of spike counts, as measured from Monte Carlo simulations; while these are in principle related to the cross-correlation functions **C**(*t*) defined in **Methods: Computing statistics from linear response theory** we will use **C**_*T*_ to denote the matrix of correlation coefficients measured for time window *T*; i.e.

$${\left(C_{T}\right)}_{ij}=\rho_{T,ij}$$ (65)

Furthermore, we will restrict to the E-E correlations; i.e. **C**_*T*_ will be a *n*_*E*_ × *n*_*E*_ matrix, with ones on the diagonal (as *ρ*_*T*,*ii*_ = 1).

When we examined the singular values of the E-E correlation matrices obtained from Monte Carlo simulations, we noticed a consistent trend: there was usually one large cluster with one positive outlier. This motivates the following simple idea: by subtracting off a multiple of the identity matrix, λ**I**, we shift the cluster towards zero; consequently **C**_*T*_ − λ**I** is close to a rank-1 matrix. We then propose to use the sum of the two as an approximation to **C**_*T*_:

$$C_{T}\approx \lambda I+\left(\sigma_{1}-\lambda \right)u_{1}u_{1}^{T}.$$ (66)

We seek the value λ which maximizes the fraction of the Frobenius norm explained by the first singular vector: i.e. in terms of the singular values,

$$\begin{array}{ccc} \lambda & = & \max_{\lambda }\frac{{\tilde{\sigma }}_{1}^{2}}{\sum_{j=1}^{r}{\tilde{\sigma }}_{j}^{2}} \end{array}$$ (67)

$$\begin{array}{ccc} & = & \max_{\lambda }\frac{{\left(\sigma_{1}-\lambda \right)}^{2}}{\sum_{j=1}^{r}{\left(\sigma_{j}-\lambda \right)}^{2}} \end{array}$$ (68)

Since **C**_*T*_ is symmetric semi-positive definite, the singular values *σ*_*j*_ are equal to the eigenvalues λ_*j*_: here *σ*_1_ ≥ *σ*_2_ ≥ ⋯ ≥ *σ*_*r*_ ≥ 0 and *r* is the rank of **C**_*T*_. This has an exact solution:

$$\begin{array}{ccc} \lambda & = & \lambda_{1}-\frac{\sum_{j>1}{\left(\lambda_{1}-\lambda_{j}\right)}^{2}}{\sum_{j>1}\lambda_{1}-\lambda_{j}} \end{array}$$ (69)

Because we have subtracted a multiple of the identity matrix, none of the singular vectors will have changed. We then have

$$C_{T}\equiv \lambda I+\left(C_{T}-\lambda I\right)$$ (70)

$$\begin{array}{ccc} & = & \lambda I+\sum_{i=1}^{r}\left(\sigma_{i}-\lambda \right)u_{i}u_{i}^{T} \end{array}$$ (71)

By truncating this sum, we approximate *C* with a shifted low-rank matrix:

$$\begin{array}{ccc} C_{T}\approx C_{T}^{\text{diag}+R1} & \equiv & \lambda I+\left(\sigma_{1}-\lambda \right)u_{1}u_{1}^{T} \end{array}$$ (72)

This procedure is similar to *factor analysis*, in which one seeks to explain a data vector as the sum of a random vector (**u**) and the linear combination of some number of latent factors (**z**) [48]:

x=Λz+u;

the entries of **x** would then have the correlation matrix Ψ + ΛΛ^*T*^, where Ψ is a diagonal matrix containing the variances of **u**.

## Supporting information

S1 Text Includes supplementary analysis of statistics and numerical methods, including discussion of supplementary figures. (PDF)

S1 Fig Theory predicts population statistics in the asynchronous regime. (TIF)

S2 Fig Theory predicts population statistics in the strong asynchronous regime. (TIF)

S3 Fig Theory predicts cell-by-cell statistics in the asynchronous regime. (TIF)

S4 Fig Theory predicts cell-by-cell statistics in the strong asynchronous regime. (TIF)

S5 Fig Theory captures low-rank structure in correlation matrices. (TIF)

S6 Fig Effective parameters in the heterogeneous network: Asynchronous regime. (TIF)

S7 Fig Effective parameters in the heterogeneous network: Strong asynchronous regime. (TIF)

S8 Fig Firing rate as a function of inhibitory conductance and threshold. (TIF)

S9 Fig Correlation increases with firing rate in the strong asynchronous regime: Erdős-Rényi networks. (TIF)

S1 Table Statistics from heterogeneous vs. homogeneous networks: Asynchronous regime. (PDF)

S2 Table Statistics in recurrent networks: Monte Carlo vs. linear response theory, asynchronous regime. (PDF)

S3 Table Statistics from heterogeneous vs. homogeneous networks: Strong asynchronous regime. (PDF)

S4 Table Statistics in recurrent networks: Monte Carlo vs. linear response theory, strong asynchronous regime. (PDF)
