# Supplementary material for: When do correlations increase with firing rates in recurrent networks?
Source: PLoS Comput Biol. 2017 Apr 27;13(4):e1005506. doi: 10.1371/journal.pcbi.1005506 (PMC5426798; doi:10.1371/journal.pcbi.1005506)
Supplement: S1 Text — (PDF) [file pcbi.1005506.s001.pdf]

Supplementary Information for
“When do Correlations Increase with Firing Rates?”

Andrea K. Barreiro and Cheng Ly

Linear response theory predicts the distribution of first- and second-order statistics in recurrent networks

In recurrent networks, the response of each cell is shaped by both direct and indirect connections through the network. To separate the impact of different network mechanisms, we applied a network linear response theory (described in **Methods: Linear Response Theory**) which allows us to decompose network correlations into contributions from different graph motifs (as in [**?**, **?**]). Here, we verify that this theory accurately predicted the results of Monte Carlo simulations.

The network connectivity matrix $\mathbf{W}$ and all other parameter values were the same as used in Monte Carlo simulations; linear response theory yields a predicted value for the stationary firing rate ν_i and spike count variance $\mathrm{Var}_T[n_i]$ of each cell i, as well as the spike count covariance of each distinct cell pair, $\mathrm{Cov}_T(n_i, n_j)$. For each distinct network, we then compared the population distribution of single cell firing rates ν_i, spike count variances, and two-cell covariances (as well as two-cell correlation coefficients ρ^{EE} and ρ^{IE}), with the population distributions we obtained from Monte Carlo simulations.

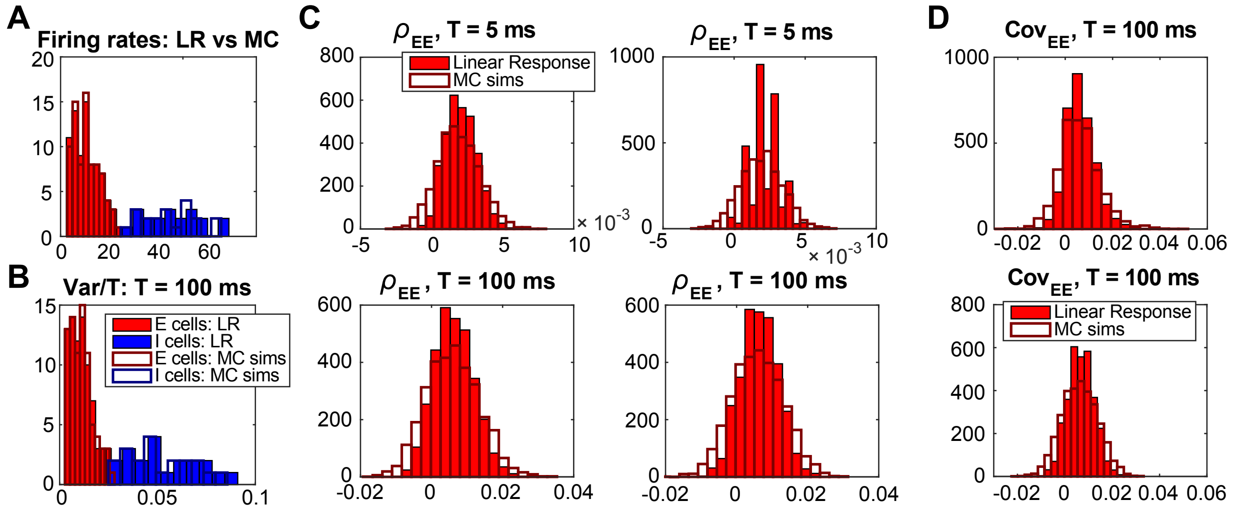

Figure S1: **Theory predicts population statistics in the asynchronous regime.** Distributions of spiking statistics, comparing the results of linear response theory with Monte Carlo simulations in the asynchronous regime. (A) Firing rates ν_i, for the heterogeneous network. (B) Normalized spike count variances, $\mathrm{Var}_T[n_i]/T$, heterogeneous network, T = 100 ms. (C) Spike count correlations, for 5 ms and 100 ms time windows: heterogeneous (left two panels) and homogeneous (right two panels). (D) Spike count *covariance*, 100 ms time windows: heterogeneous (top) and homogeneous (bottom).

We first consider the firing rate, shown in Fig. S1A. In the heterogeneous network, both excitatory and inhibitory firing rates have large ranges that span approximately an order of magnitude.

The linear response theory accurately captures all aspects of the firing rate distributions. The inhibitory firing rates are higher than excitatory, consistent with this population receiving a stronger excitatory input (vs. the excitatory population; compare W_{IE}/N_E and W_{EE}/N_E, from Table 1 in the main text). In the homogeneous network, firing rates are strongly clustered around their mean values (shown in Table S1). They are also well-predicted, although the linear response theory does appear to slightly overestimate the inhibitory rates (see Table S2). Similarly, spike count variances match well (shown for long time windows (T = 100 ms) in Fig. S1B).

Table S1: **Statistics from heterogeneous vs. homogeneous networks: asynchronous regime**

	Heterogenous		Homogenous	
Statistic	E	I	E	I
Firing rate (Hz)	10.6 ± 5.0	44.3 ± 11.3	10.1 ± 0.046	43.5 ± 0.37
Var_T, T = 5 ms	0.051 ± 0.023	0.19 ± 0.048	0.048 ± 0.0002	0.19 ± 0.0014
Var_T, T = 100 ms	1.14 ± 0.58	5.16 ± 1.67	1.06 ± 0.0095	4.99 ± 0.096
	Heterogenous		Homogenous	
ρ^{EE}, T = 5 ms	0.0019 ± 0.0015		0.0019 ± 0.0015	
ρ^{EE}, T = 50 ms	0.0058 ± 0.0062		0.0060 ± 0.0059	
ρ^{EE}, T = 100 ms	0.0059 ± 0.0075		0.0059 ± 0.0072	

Firing statistics from Monte Carlo simulations of recurrent networks in the asynchronous regime.

We now consider a common measure of noise correlations, the spike count (Pearson's) correlation of pairs of excitatory cells in a particular time window (Eqn. (40), main text). As in the Monte Carlo simulations, we have assumed spike count statistics to be stationary over time, so that for each T, spike counts n_i and n_j are treated as random variables sampled both over realizations (i.e trials) and time t. We computed these statistics for both short (T = 5 ms) and long (T = 100 ms) time windows, and illustrate them in Figure S1C; statistics from both heterogeneous (left panels) and homogenous (right panels) are show. E-E correlations are weakly positive, with a small fraction of pairs (~ 5%) having values below zero. In all panels, the mean/median of the distribution are captured well by the linear response theory; however, the linear response calculation appears to slightly underestimate the simulated variance, as evidenced by the "taller and thinner" distribution shown in solid red (each histogram is computed by distributing 80 × 79/2 distinct coefficients over equally sized bins). The correlation values computed by linear response have comparable ranges in the heterogeneous and homogenous networks, similar to MC simulations and in contrast to first-order statistics.

Linear response theory also predicts the distribution of spike count *covariances* (i.e. the numerator of Eqn. (40)): we show these in Fig. S1D. As for correlations, theory underestimates the observed variance of the distributions. However, it appears to capture the fat right tails in the heterogeneous network very well (Fig. S1D, top row).

We now turn our attention to the *strong asynchronous* (**SA**) regime, in which both types of excitatory connections were strengthened (see Table 1); the resulting network shows occasional, irregular bursts of concentrated activity (see Fig. 1B). Many of the overall trends are similar to the asynchronous case; we focus on the differences.

Excitatory firing rates were slightly under-predicted by linear response theory (Fig. S2A; see

Table S2: **Statistics in recurrent networks: Monte Carlo vs. linear response theory, asynchronous regime**

	Heterogenous				Homogenous			
	μ		σ		μ		σ	
Statistic	MC	LR	MC	LR	MC	LR	MC	LR
Firing rate, E	10.6	10.6	5.0	5.3	10.1	10.0	4.6×10^{-2}	2.3×10^{-2}
Firing rate, I	44.3	45.9	11.3	12.0	43.5	45.0	0.37	0.32
FF, 5 ms, E	0.9585	0.9647	0.0148	0.0162	0.9576	0.9640	4.53×10^{-4}	1.12×10^{-4}
FF, 5 ms, I	0.8725	0.8726	0.0093	0.0091	0.8690	0.8688	9.4×10^{-4}	5.81×10^{-4}
FF, 100 ms, E	1.0573	1.0587	0.0345	0.0305	1.0493	1.0504	0.0074	0.0024
FF, 100 ms, I	1.1449	1.1540	0.0810	0.0859	1.1460	1.1528	0.0164	0.0099
ρ^{EE}, 5 ms ($\times 10^{-3}$)	1.9	2.0	1.5	1.1	1.9	2.1	1.5	1.0
ρ^{EE}, 50 ms ($\times 10^{-3}$)	5.8	6.3	6.2	4.8	6.0	6.4	5.9	4.6
ρ^{EE}, 100 ms ($\times 10^{-3}$)	5.9	6.3	7.5	5.3	5.9	6.4	7.2	5.3

Comparing Monte Carlo simulations with predictions from linear response; firing statistics in the asynchronous regime. Statistics displayed here are: firing rates for both excitatory and inhibitory populations; Fano factor (FF) for both excitatory and inhibitory populations; spike count correlations for excitatory pairs only (ρ^{EE}). Standard deviations are reported across the population; i.e. across eighty (80) E cells, or twenty (20) I cells, or 3160 E-E pairs.

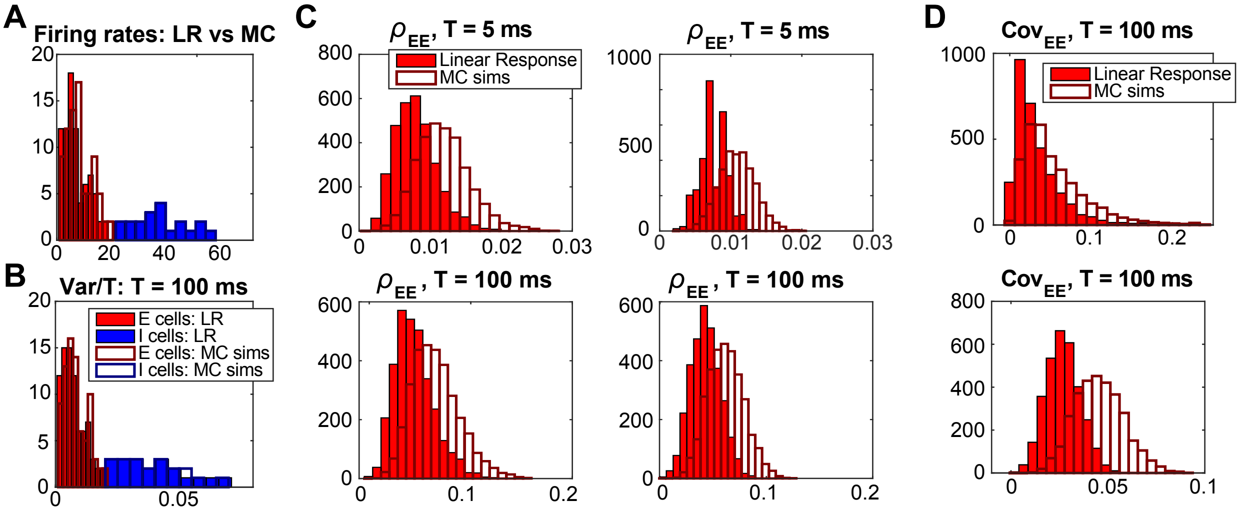

Figure S2: **Theory predicts population statistics in the strong asynchronous regime.** Distributions of spiking statistics, comparing the results of linear response theory with Monte Carlo simulations in the *strong asynchronous* regime. (A) Firing rates ν_i, for the heterogeneous network. (B) Normalized spike count variances, $\mathrm{Var}_T[n_i]/T$, heterogeneous network, T = 100 ms. (C) Spike count correlations, for 5 ms and 100 ms time windows: heterogeneous (left two panels) and homogeneous (right two panels). (D) Spike count *covariance*, 100 ms time windows: heterogeneous (top) and homogeneous (bottom).

Table S3: **Statistics from heterogeneous vs. homogeneous networks: strong asynchronous regime**

	Heterogenous		Homogenous	
Statistic	E	I	E	I
Firing rate (Hz)	8.1 ± 4.5	36.6 ± 9.8	7.2 ± 0.095	35.2 ± 0.41
Var_T, $T = 5$ ms	0.039 ± 0.021	0.16 ± 0.040	0.035 ± 0.0004	0.15 ± 0.0016
Var_T, $T = 100$ ms	0.84 ± 0.48	3.93 ± 1.31	0.74 ± 0.013	3.75 ± 0.079
	Heterogenous		Homogenous	
ρ^{EE}, $T = 5$ ms	0.0119 ± 0.0037		0.0109 ± 0.0025	
ρ^{EE}, $T = 50$ ms	0.0622 ± 0.0206		0.0587 ± 0.0147	
ρ^{EE}, $T = 100$ ms	0.0654 ± 0.0232		0.0618 ± 0.0169	

Firing statistics from Monte Carlo simulations of recurrent networks in the strong asynchronous regime.

Table S3 for homogeneous rates). Similarly, spike count variances (Fig. S2B) were under predicted. Spike count correlations ρ^{EE} are now positive, with few or no negative correlations (Fig. S2C). The mean is significantly under-predicted; the predicted distributions appear slightly narrower than the observed (Monte Carlo) distribution. Spike count covariances for long time windows are shown in Fig. S2D; the linear response theory appears to capture the qualitative shape of the distributions, particularly the fat right tail in the heterogeneous network (top panel). However, as for correlations (Fig. S2C), the mean is under-predicted in both networks (Table S4).

Linear response theory predicts the first- and second-order statistics of individual cells

We next investigate how well these statistics are predicted on a *cell-to-cell* basis. This is crucially important when individual correlation coefficients ρ_{ij} within a simulation may vary over an order of magnitude or even in sign. For example, consider the heterogeneous network illustrated in Fig. 2C(bottom): E-E correlations were weakly positive (on average less than 0.01) but could range as high as 0.03 or as negative as -0.015 for some cell pairs. If I pick a specific cell pair i, j out of the population, can I predict where in this range ρ_{ij} will fall? Predicting the correlation of specific cell pairs would be a valuable tool, as many models for heterogeneity — such as those based on population density methods (e.g. [**?**]) — do not capture cell-to-cell variation.

We find that single-cell statistics are very accurately predicted for the heterogeneous network. In Figure S3, we show firing rate (Fig. S3A) and Fano factor (Fig. S3B) for three time windows: $T = 5, 50,$ 100 ms. In each panel, both quantities from the Monte Carlo simulations and linear response theory are plotted, on a cell-by-cell basis. In Fig. S3A, the red stars give the firing rate of the uncoupled neurons (i.e. determined only by the threshold θ_i and the level of background noise). The effect of coupling is to lower the firing rate of the E cells but to raise the firing rate of the I cells; this is captured very well by the fixed point iteration of Eqn. (22). There is still significant heterogeneity in the firing rates due to variable threshold, with high threshold neurons maintaining comparatively lower firing rates and low threshold neurons maintaining comparatively higher firing rates.

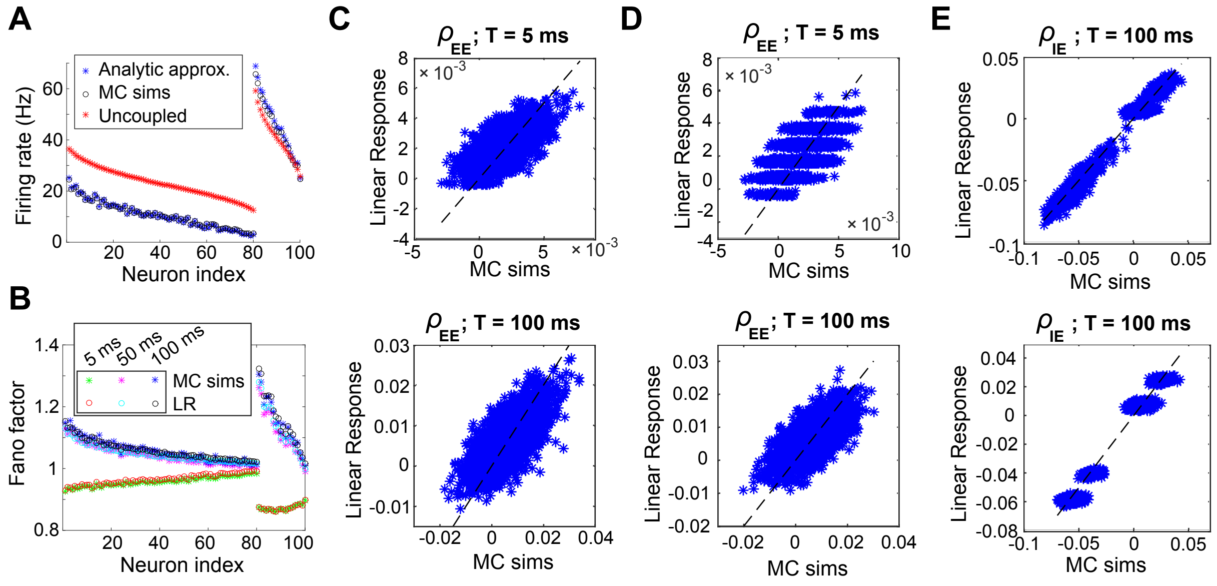

Figure S3: **Theory predicts cell-by-cell statistics in the asynchronous regime.** Distributions of spiking statistics, comparing the results of linear response theory with Monte Carlo simulations in the asynchronous regime, cell-by-cell. (A) Firing rates ν_i, for the heterogeneous network. (B) Fano factor, heterogeneous network. Time windows (T) shown are: 5 ms, 50 ms, 100 ms. (C) Spike count correlations, heterogeneous network. Time windows are: 5 ms (top) and 100 ms (bottom). (D) Spike count correlations, homogenous network. Time windows are: 5 ms (top) and 100 ms (bottom). (E) E-I spike count correlations, T = 100: heterogenous (top) and homogeneous (bottom).

Table S4: **Statistics in recurrent networks: Monte Carlo vs. linear response theory, strong asynchronous regime**

	Heterogenous				Homogenous			
	μ		σ		μ		σ	
Statistic	MC	LR	MC	LR	MC	LR	MC	LR
Firing rate, E	8.14	6.84	4.5	4.2	7.2	6.0	0.095	0.057
Firing rate, I	36.6	36.4	9.8	9.9	35.2	34.9	0.41	0.36
FF, 5 ms, E	0.9622	0.9829	0.0190	0.0155	0.9653	0.9853	4.80×10^{-4}	2.69×10^{-4}
FF, 5 ms, I	0.8719	0.8802	0.0147	0.0136	0.8709	0.8788	0.0014	0.0012
FF, 100 ms, E	1.0271	1.0578	0.0226	0.0206	1.0216	1.0516	0.0116	0.0059
FF, 100 ms, I	1.0581	1.0881	0.0698	0.0733	1.0655	1.0948	0.0124	0.0118
ρ^{EE}, 5 ms ($\times 10^{-3}$)	11.9	8.0	3.7	2.9	10.9	7.6	2.5	1.9
ρ^{EE}, 50 ms ($\times 10^{-3}$)	62.2	41.9	20.6	17.2	58.7	40.3	14.7	12.0
ρ^{EE}, 100 ms ($\times 10^{-3}$)	65.4	44.2	23.2	19.4	61.8	42.8	16.9	14.0

Comparing Monte Carlo simulations with predictions from linear response; firing statistics in the strong asynchronous regime. Statistics displayed here are: firing rates for both excitatory and inhibitory populations; Fano factor (FF) for both excitatory and inhibitory populations; spike count correlations for excitatory pairs only (ρ^{EE}). Standard deviations are reported across the population; i.e. across eighty (80) E cells, or twenty (20) I cells, or 3160 E-E pairs.

We now analyze the ability of linear response to predict *two-cell* statistics. In Fig. S3C we plot the spike count correlation $\rho_{ij} = \mathrm{Cov}_T(n_i, n_j)/\sqrt{\mathrm{Var}[n_i]\,\mathrm{Var}[n_j]}$, for all possible E-E pairs in the heterogeneous network, at both $T = 5$ ms (top) and 100 ms (bottom). The values predicted by linear response theory matches well with the Monte Carlo simulations in both overall range and cell-to-cell; in both plots, the points cluster around the unity line.

We now consider how well linear response models the homogeneous network on a cell-to-cell basis. As in the heterogeneous network, single-cell statistics are accurately predicted (because both simulated and predicted single-cell statistics are nearly constant across the population, we report their values in Table S2). Firing rate is slightly overestimated, as is variance. Fano factor differs systemically with time interval: cell activities appear slightly "sub-Poisson" for $T = 5$ ms, but "super-Poisson" for $T = 50, 100$ ms. We then examined two-cell statistics: E-E correlations were weak and positive, and clustered in a cloud around the unity line (Fig. S3D), for both short (T =5 ms, top) and long (T =100 ms, bottom) time windows.

Although we mostly focus on E-E correlations here, we observed excellent results in predicting other two-cell statistics, for example excitatory-inhibitory (E-I) correlations. In Fig. S3E we show E-I correlations for $T = 100$ ms, for both the heterogeneous (top) and homogeneous (bottom) networks. E-I correlations took on a wider range of values; both positive and negative, with a range between $[-0.15, 0.15]$ for $T = 100$ ms. In the homogeneous network they cluster in four distinct clouds (Fig. S3E, bottom): on closer inspection, these correspond to the presence or absence of direct connections between the pairs. For E-I pairs with no direct connection, correlations are weak and positive. Pairs with only a $E \to I$ connection are strongly positively correlated, while pairs with only an $I \to E$ connection are strongly negatively correlated. Pairs with BOTH connections are weakly negatively correlated, which may reflect the fact that $W_{IE} > W_{EI}$. We also find good

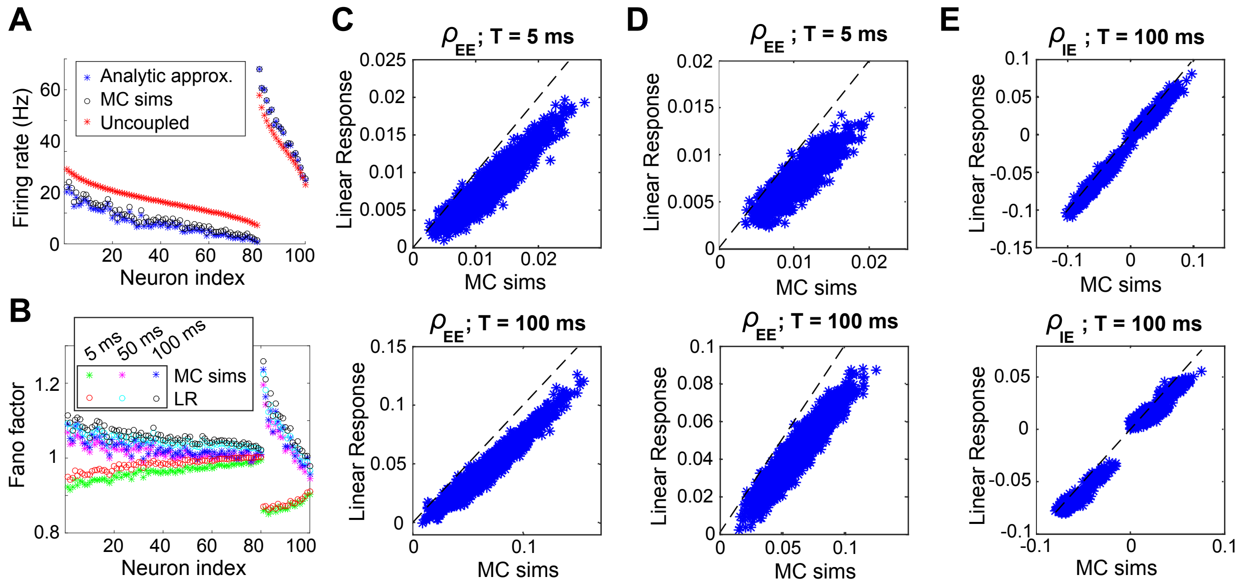

Figure S4: **Theory predicts cell-by-cell statistics in the strong asynchronous regime.** Comparing the results of linear response theory with Monte Carlo simulations in the *strong asynchronous* regime, cell-by-cell. (A) Firing rates ν_i, for the heterogeneous network. (B) Fano factor, heterogeneous network. Time windows (T) shown are: 5 ms, 50 ms, 100 ms. (C) Spike count correlations, heterogeneous network. Time windows are: 5 ms (top) and 100 ms (bottom). (D) Spike count correlations, homogenous network. Time windows are: 5 ms (top) and 100 ms (bottom). (E) E-I spike count correlations, $T = 100$: heterogenous (top) and homogeneous (bottom).

results when we move to the strongly asynchronous case. This network has increased excitation ($W_{EE} = 9$ and $W_{EI} = 8$, vs. $W_{EE} = 5$ and $W_{EI} = 0.5$ in the asynchronous regime) and shows short bursts of activity (see Fig. 1); since this violates the assumption of constant firing rate, *a priori* we cannot be sure linear response theory will be successful. However, the theory is nonetheless successful at matching broad trends in firing rate, Fano factors, and cell-pair correlations (Fig. S4). There are differences between the simulations and linear response calculations. For excitatory neurons, firing rate is slightly overestimated (Fig. S4A), variance underestimated and Fano factor overestimated (Fig. S4B). For inhibitory neurons, firing rate appears to be very accurate; variance and Fano factor are slightly overestimated. We also see that ρ^{EE} is systematically underestimated (Fig. S4C, heterogeneous; Fig. S4D, homogeneous;); ρ^{IE} may also be slightly underestimated, but less so (see Fig. S4E).

Finally, the cell-by-cell accuracy of the linear response theory is reflected in the overall structure of the correlation matrix. We performed the diagonal plus rank-one analysis on correlation matrices we obtained from linear response theory (Fig. S5). We see the same patterns observed in Fig 4; in the strong asynchronous regime there is a strong positive relationship with firing rate, which is reflected in the weights of the first singular vector.

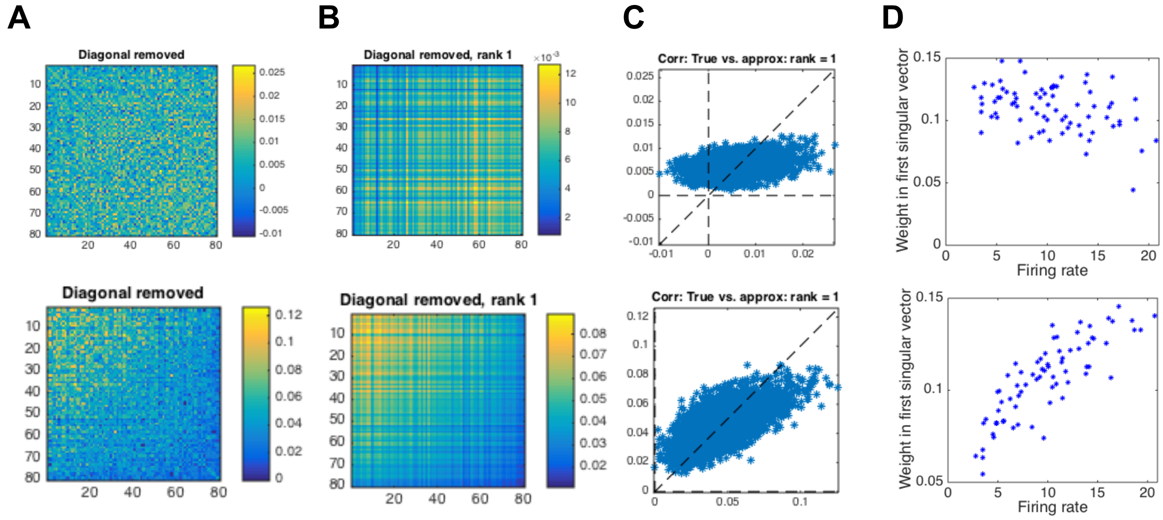

Figure S5: **Theory captures low-rank structure in correlation matrices.** Approximating correlation matrices (for the heterogeneous networks) obtained from linear response theory, as a diagonal plus rank-one. In each column of (A-D), the asynchronous (top) and strong asynchronous (bottom) regimes are shown; T = 100 ms. (A) The shifted E-E correlation matrix, $\mathbf{C}_T - \lambda\mathbf{I}$, for an appropriately chosen λ. (B) A rank-one approximation to $\mathbf{C}_T - \lambda\mathbf{I}$. (C) True correlation coefficients vs. rank-one approximation, cell-by-cell. (D) Weight in the first singular vector, $\mathbf{u}_1$ vs.geometric mean firing rate $\sqrt{\nu_i \nu_j}$.

Approximating single-cell susceptibility in a heterogeneous network

To understand how the single-cell susceptibility (Eqn. (4), main text) depends on the six parameters $\langle g_{I,i} \rangle$, etc., we plotted each parameter vs. firing rate (see Fig. S6 and Fig. S7 for the asynchronous and strong asynchronous regimes respectively). The panels in Fig. S6A show $\langle g_{I,i} \rangle$, $\sigma_{I,i}$, $\langle g_{E,i} \rangle$, and $\sigma_{E,i}$, which appear to be randomly scattered with no relationship to firing rate (there is also no apparent relationship in the derived relationships for effective time constant $\tau_{\text{eff},i}$, effective potential μ_i, and effective noise $\sigma_{\text{eff},i}$, Fig. S6B); σ_i is constant for all cells. However, for both networks there is a clear relationship with θ_i. Furthermore, of the four non-constant parameters with no discernible relationship, the values of $\langle g_I \rangle$ appear to have the greatest spread; we therefore hypothesized that we can approximate $S_i^{\langle g_I \rangle}$, by reevaluating the firing rate function in which $\sigma_{I,i}$, $\langle g_{E,i} \rangle$, $\sigma_{E,i}$ and σ_i have been replaced by their average values: i.e.

$$\hat{S}_i^{\langle g_I \rangle} \equiv \frac{1}{\sqrt{F(\langle g_{I,i} \rangle, \theta_i)}} \frac{\partial F}{\partial x_1} (\langle g_{I,i} \rangle, \theta_i) \quad (1)$$

where

$$F(\langle g_{I,i} \rangle, \theta_i) \equiv f(\langle g_{I,i} \rangle, \langle \sigma_{I,i} \rangle_p, \langle \langle g_{E,i} \rangle \rangle_p, \langle \sigma_{E,i} \rangle_p, \langle \sigma_i \rangle_p, \theta_i) \quad (2)$$

and $\langle \cdot \rangle_p$ denotes the population average.

To explore the role of how the *cause* of firing rate diversity might regulate correlations, we next plot firing rate as a function of threshold θ and mean inhibitory conductance $\langle g_I \rangle$; that is, we plot $F(\langle g_I \rangle, \theta)$ (see Eqn. (2)). In both regimes, firing rate decreases from left to right and bottom to top (Fig. S8). Any curve transversal to the level curves of the firing rate will sample a wide range of firing rates, but possibly different susceptibilities. The points corresponding to the actual excitatory cells in our network are illustrated in red; black squares illustrate an alternate curve, where $\langle g_I \rangle$ is varied but $\theta = 1$.

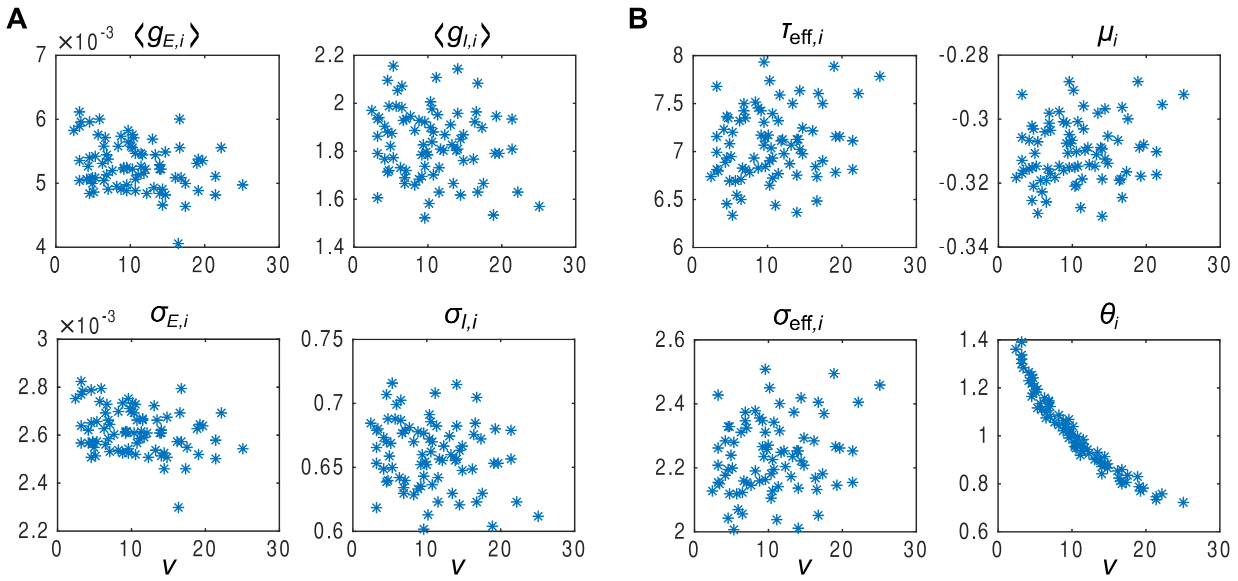

Figure S6: **Effective parameters in the heterogeneous network: asynchronous regime** Parameters used to estimate susceptibility, for all excitatory neurons in the network. Each parameter is plotted vs. firing rate. (A) Mean excitatory conductance $\langle g_{E,i} \rangle$ (top left), mean inhibitory conductance $\langle g_{I,i} \rangle$ (top right), excitatory conductance variability $\sigma_{E,i}$ (bottom left), and inhibitory conductance variability $\sigma_{I,i}$ (bottom right). (B) Effective time constant $\tau_{\text{eff},i}$ (top left), effective input current μ_i (top right), effective current noise variability $\sigma_{\text{eff},i}$ (bottom left), and threshold θ_i (bottom right).

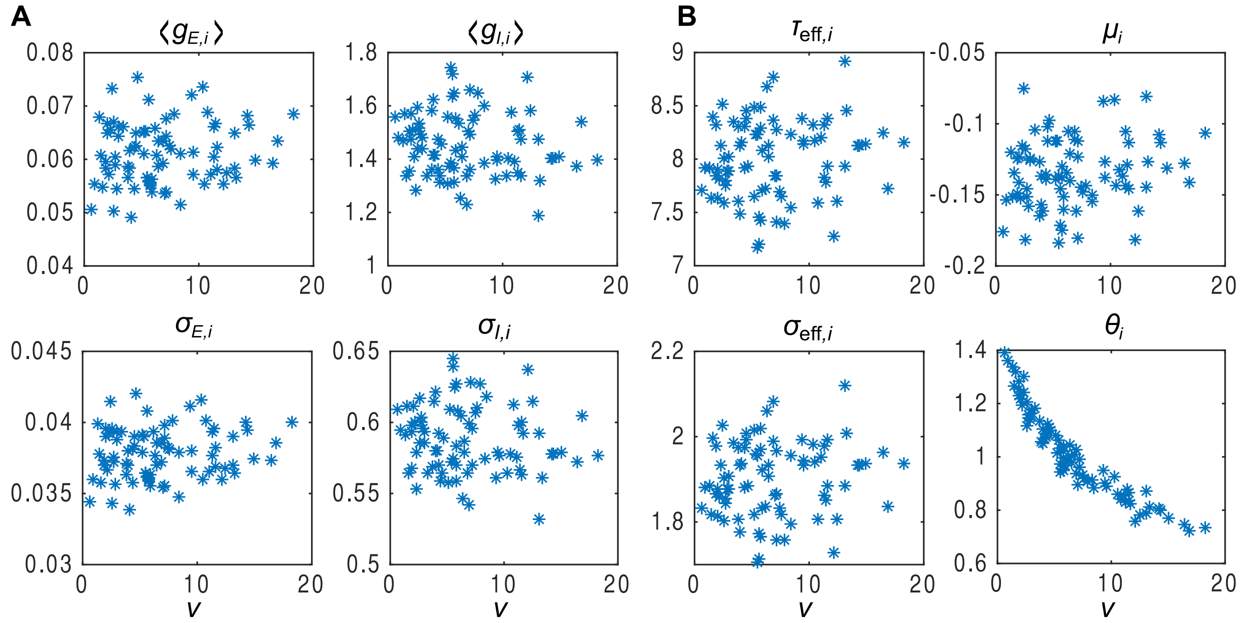

Figure S7: **Effective parameters in the heterogeneous network: strong asynchronous regime** Parameters used to estimate susceptibility, for all excitatory neurons in the network. Each parameter is plotted vs. firing rate. (A) Mean excitatory conductance $\langle g_{E,i} \rangle$ (top left), mean inhibitory conductance $\langle g_{I,i} \rangle$ (top right), excitatory conductance variability $\sigma_{E,i}$ (bottom left), and inhibitory conductance variability $\sigma_{I,i}$ (bottom right). (B) Effective time constant $\tau_{\text{eff},i}$ (top left), effective input current μ_i (top right), effective current noise variability $\sigma_{\text{eff},i}$ (bottom left), and threshold θ_i (bottom right).

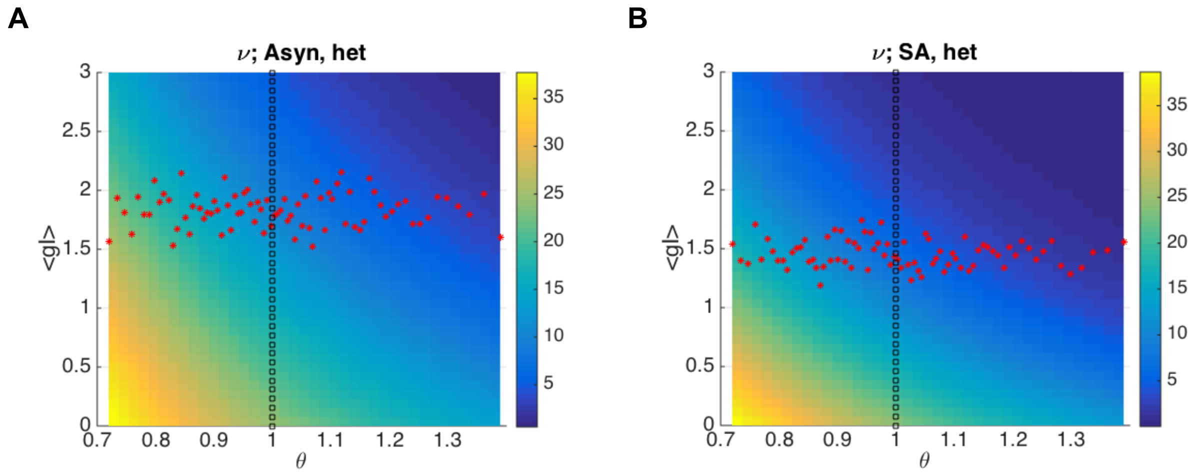

Figure S8: **Firing rate as a function of inhibitory conductance and threshold.** Firing rate of a conductance-based LIF neuron, as a function of mean inhibitory conductance $\langle g_I \rangle$ and threshold θ: $\hat{S}^{\langle g_I \rangle}(\langle g_I \rangle, \theta)$ (defined in Eqn (19)). Other parameters are set to the population average. Overlays show $(\langle g_{I,i} \rangle, \theta_i)$ values of the actual cells in the network (red stars) and an alternative curve through the plane, $(\langle g_I \rangle, 1)$, along which comparable firing rate diversity can be observed (black squares). (A) Asynchronous regime. (B) Strong asynchronous regime.

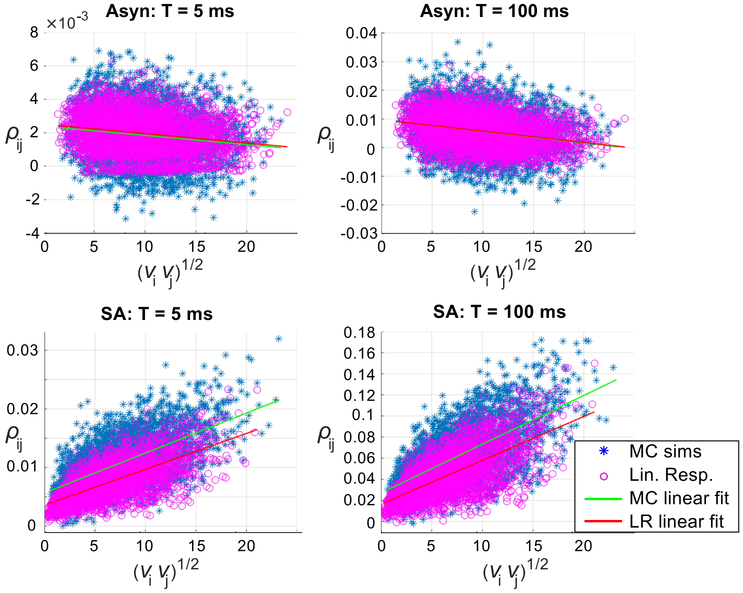

Figure S9: **Correlation increases with firing rate in the strong asynchronous regime: Erdős-Rényi networks.** E-E correlation ρ_{ij} vs. geometric mean firing rate $\sqrt{\nu_i \nu_j}$, cell-by-cell comparison of Monte Carlo simulations (blue stars) and linear response (magenta circles), in a heterogeneous network. As Fig. 2 in the main text, except that each network connection was chosen independently with a probability that depended only on E/I identity. All other parameters, including connection probabilities, were as described in the Methods. Left to right: time window T = 5 ms and 100 ms. Top row: asynchronous regime. Bottom row: strong asynchronous regime
