# Supplementary figures and images for: When do correlations increase with firing rates in recurrent networks?
Source: PLoS Comput Biol. 2017 Apr 27;13(4):e1005506. doi: 10.1371/journal.pcbi.1005506 (PMC5426798; doi:10.1371/journal.pcbi.1005506)

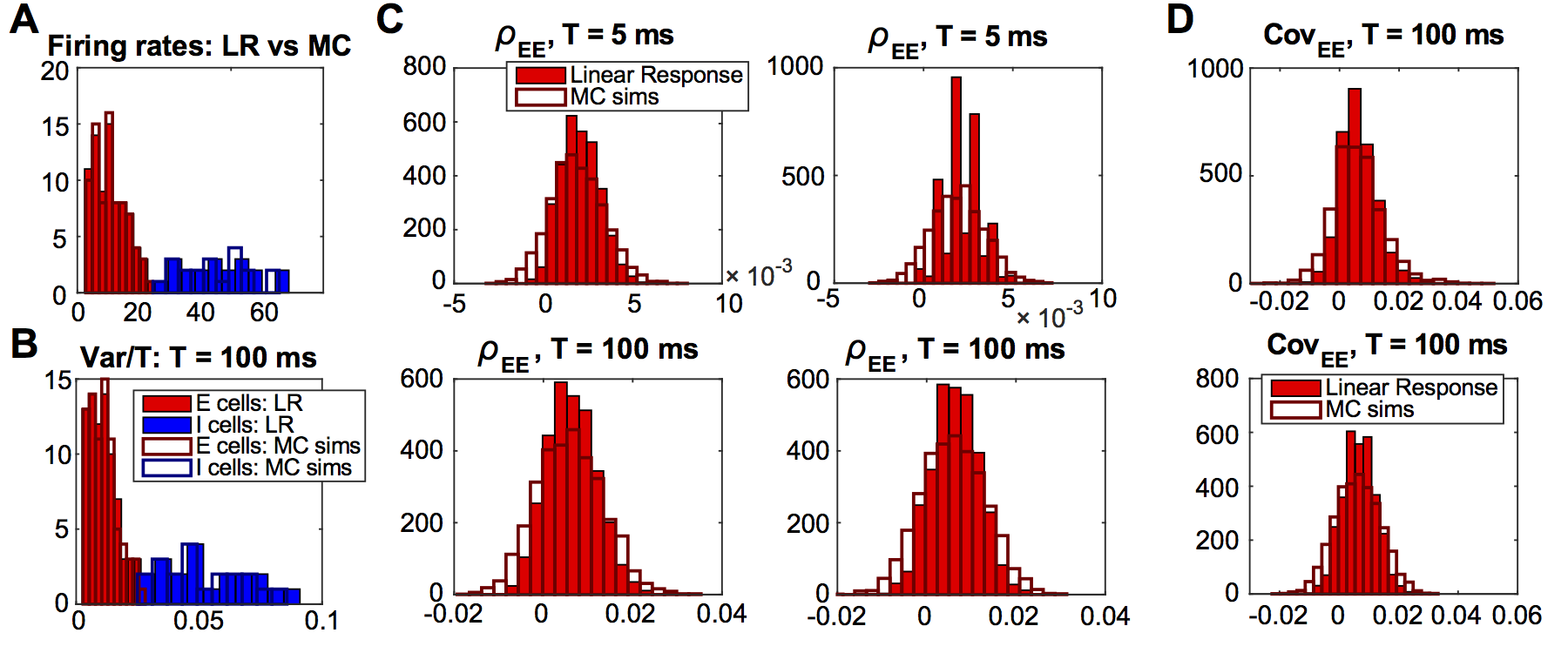

Supplement: S1 Fig — (TIF) [file pcbi.1005506.s002.tif]

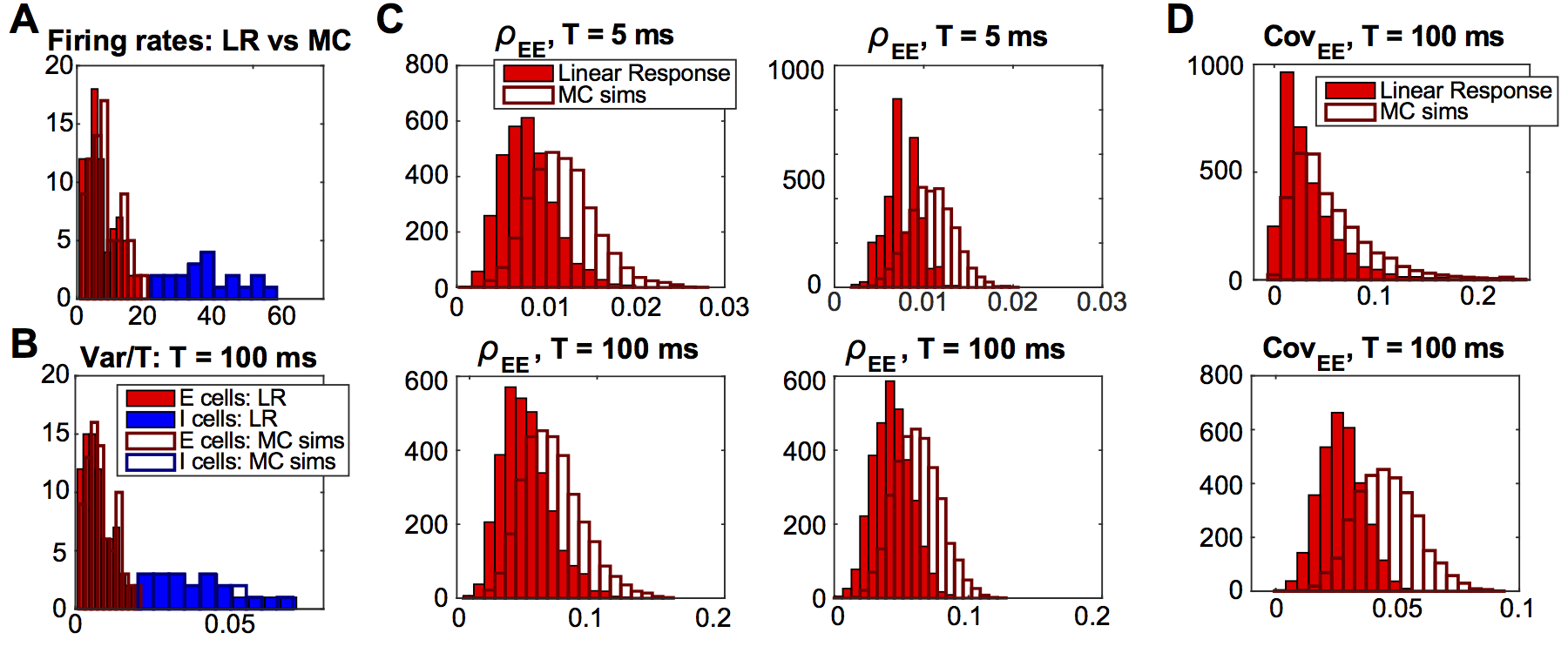

Supplement: S2 Fig — (TIF) [file pcbi.1005506.s003.tif]

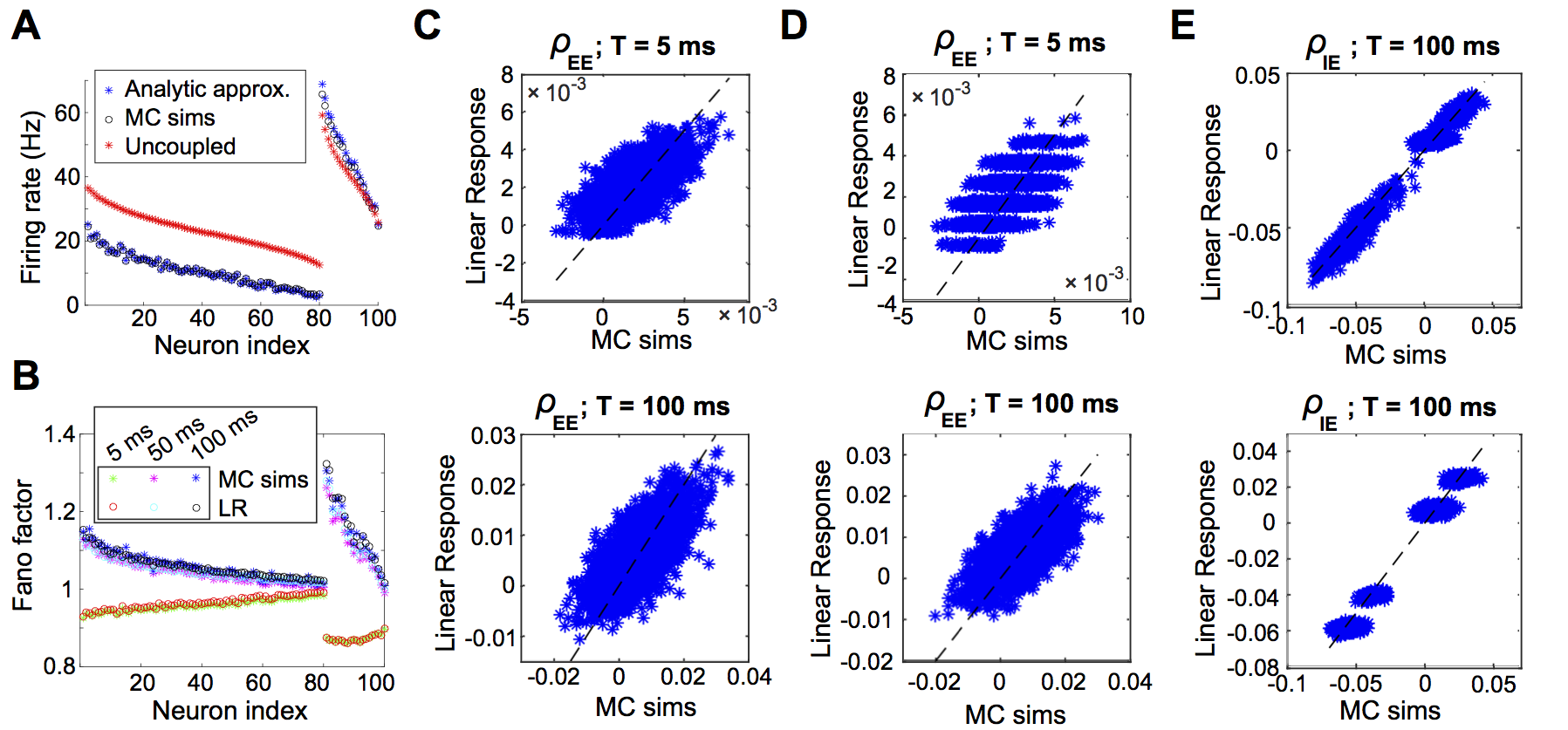

Supplement: S3 Fig — (TIF) [file pcbi.1005506.s004.tif]

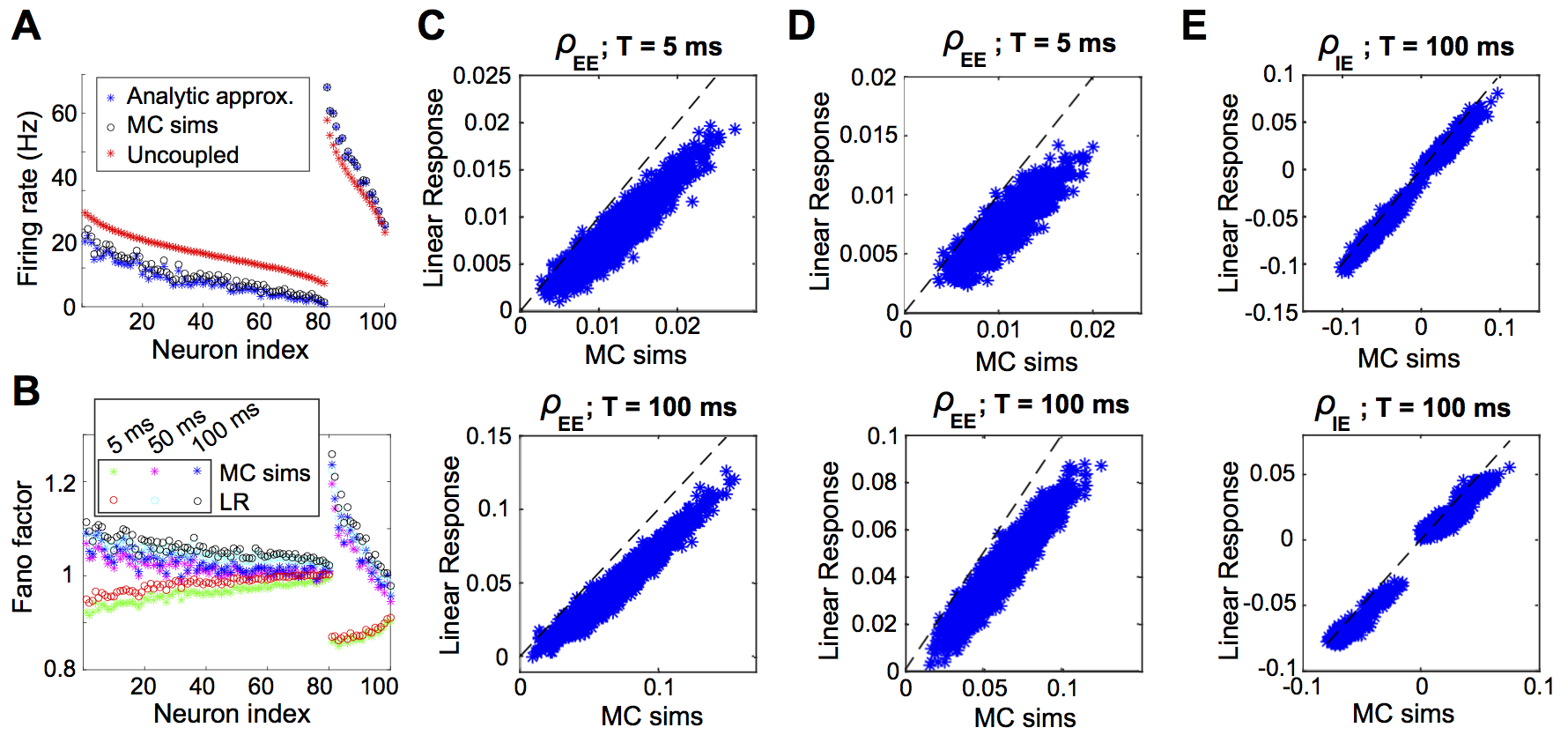

Supplement: S4 Fig — (TIF) [file pcbi.1005506.s005.tif]

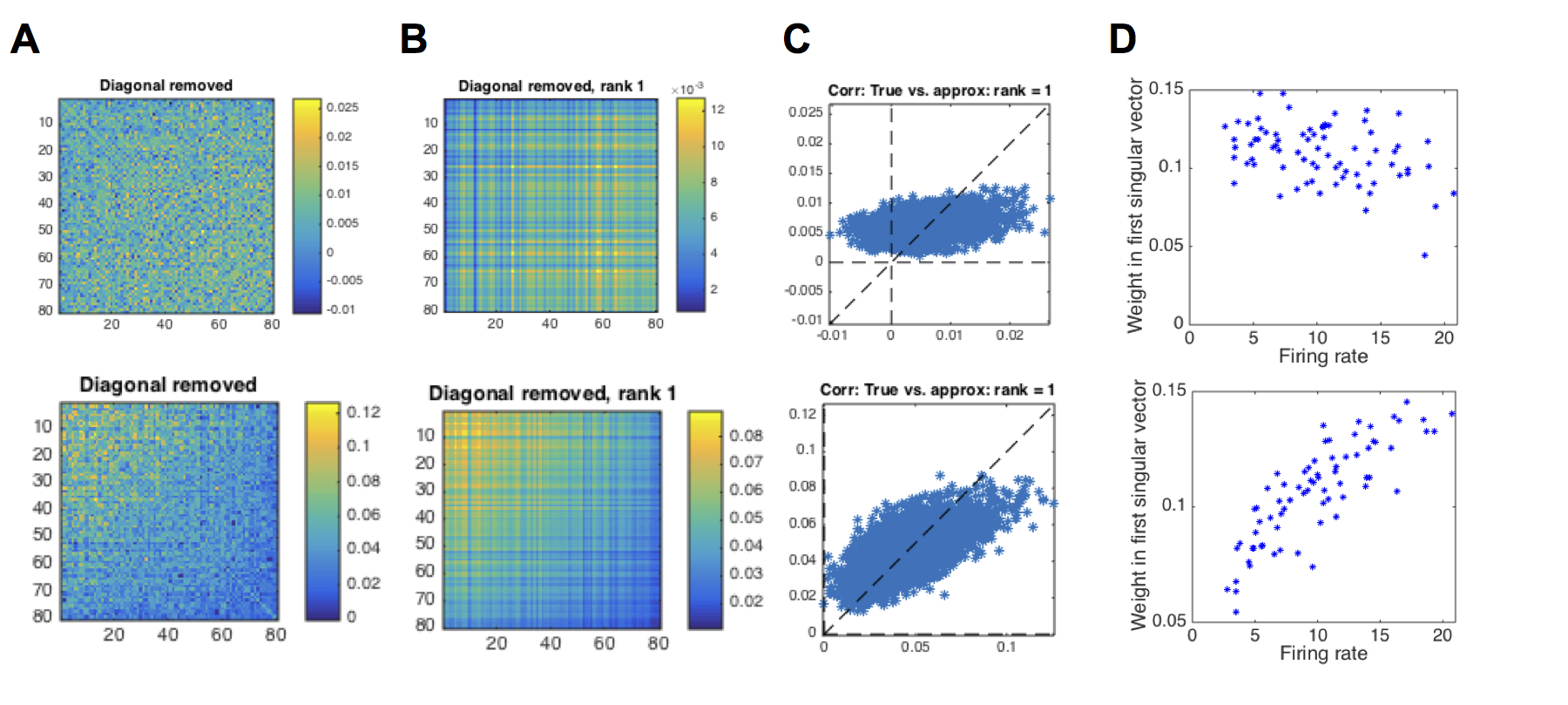

Supplement: S5 Fig — (TIF) [file pcbi.1005506.s006.tif]

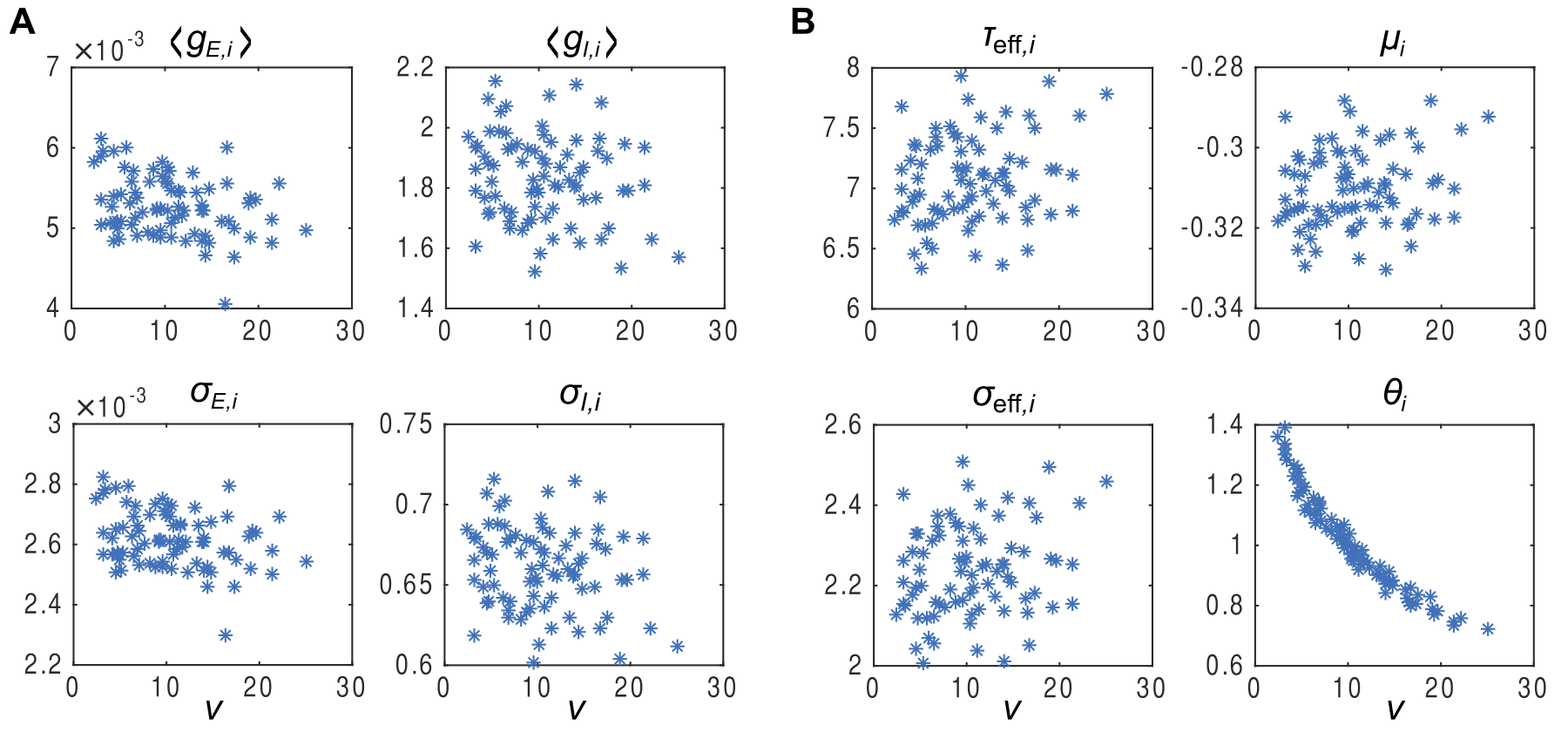

Supplement: S6 Fig — (TIF) [file pcbi.1005506.s007.tif]

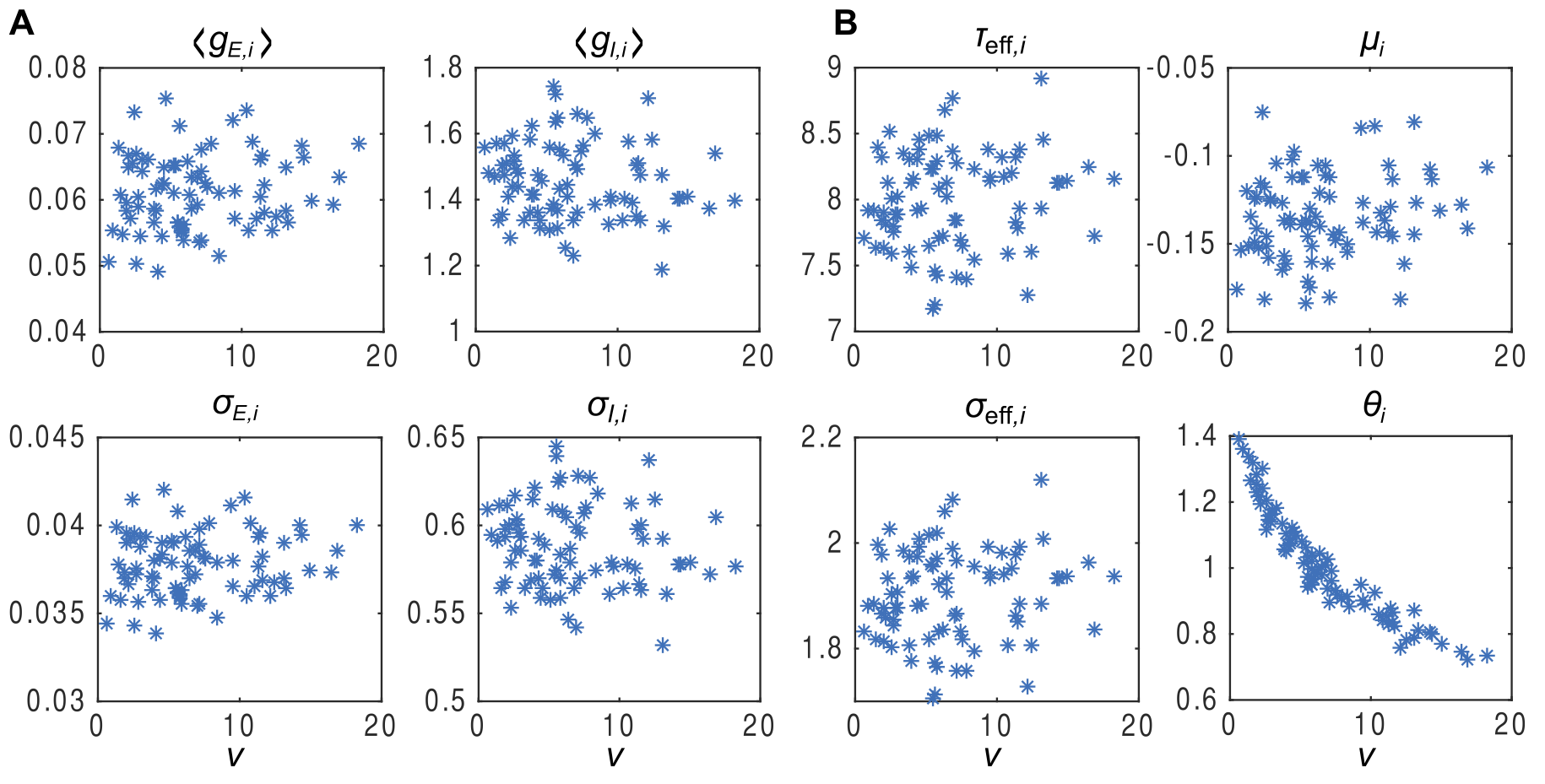

Supplement: S7 Fig — (TIF) [file pcbi.1005506.s008.tif]

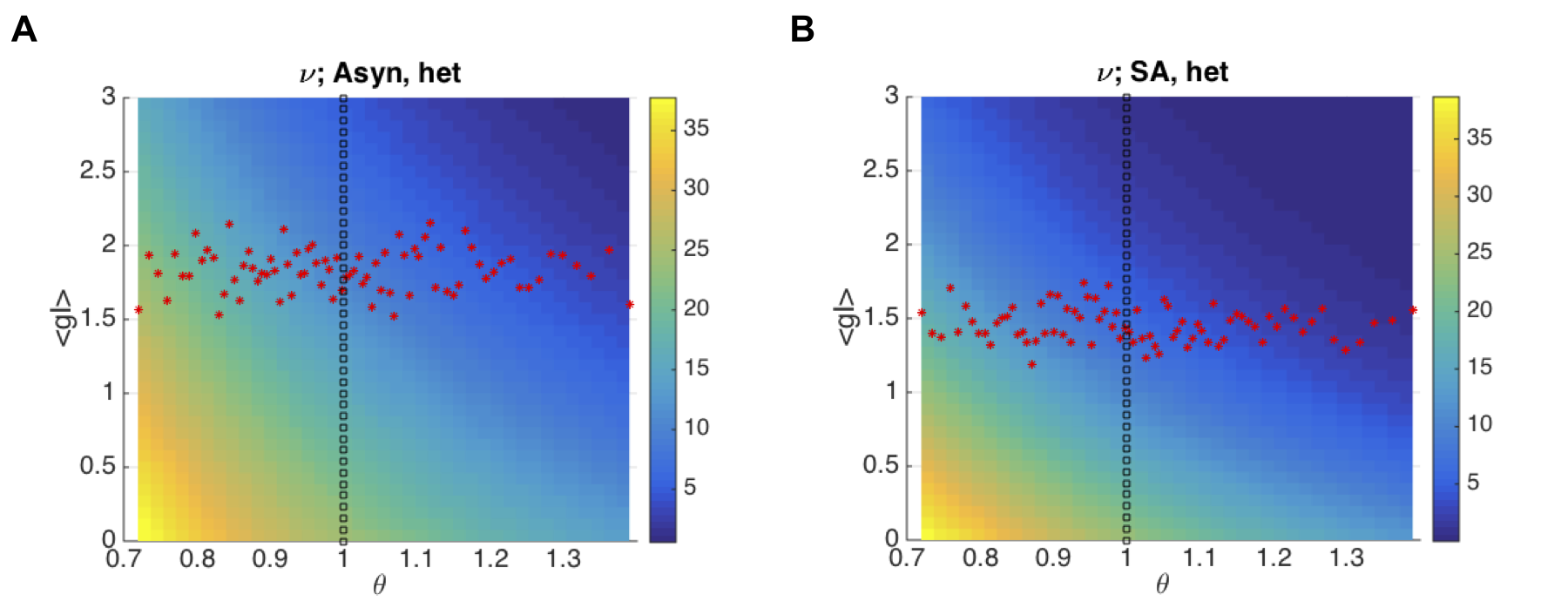

Supplement: S8 Fig — (TIF) [file pcbi.1005506.s009.tif]

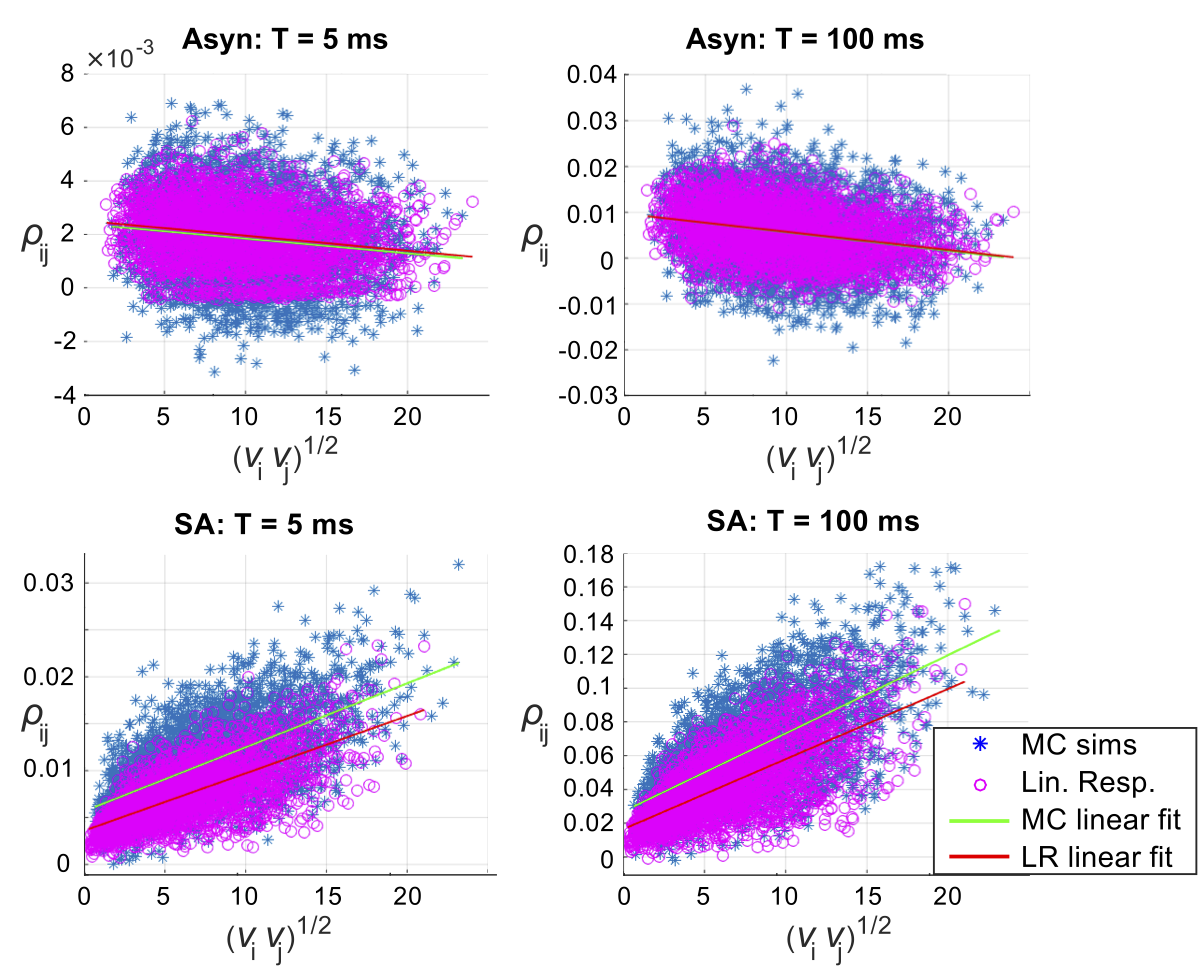

Supplement: S9 Fig — (TIF) [file pcbi.1005506.s010.tif]
