# Supplementary material for: When do correlations increase with firing rates in recurrent networks?
Source: PLoS Comput Biol. 2017 Apr 27;13(4):e1005506. doi: 10.1371/journal.pcbi.1005506 (PMC5426798; doi:10.1371/journal.pcbi.1005506)
Supplement: S1 Table — (PDF) [file pcbi.1005506.s011.pdf]

Table S1: **Statistics from heterogeneous vs. homogeneous networks: asynchronous regime**

	Heterogenous		Homogenous	
Statistic	E	I	E	I
Firing rate (Hz)	10.6 ± 5.0	44.3 ± 11.3	10.1 ± 0.046	43.5 ± 0.37
Var_T, T = 5 ms	0.051 ± 0.023	0.19 ± 0.048	0.048 ± 0.0002	0.19 ± 0.0014
Var_T, T = 100 ms	1.14 ± 0.58	5.16 ± 1.67	1.06 ± 0.0095	4.99 ± 0.096
	Heterogenous		Homogenous	
ρ^{EE}, T = 5 ms	0.0019 ± 0.0015		0.0019 ± 0.0015	
ρ^{EE}, T = 50 ms	0.0058 ± 0.0062		0.0060 ± 0.0059	
ρ^{EE}, T = 100 ms	0.0059 ± 0.0075		0.0059 ± 0.0072	

Firing statistics from Monte Carlo simulations of recurrent networks in the asynchronous regime.
