# Supplementary material for: When do correlations increase with firing rates in recurrent networks?
Source: PLoS Comput Biol. 2017 Apr 27;13(4):e1005506. doi: 10.1371/journal.pcbi.1005506 (PMC5426798; doi:10.1371/journal.pcbi.1005506)
Supplement: S2 Table — (PDF) [file pcbi.1005506.s012.pdf]

Table S2: **Statistics in recurrent networks: Monte Carlo vs. linear response theory, asynchronous regime**

	Heterogenous				Homogenous			
	μ		σ		μ		σ	
Statistic	MC	LR	MC	LR	MC	LR	MC	LR
Firing rate, E	10.6	10.6	5.0	5.3	10.1	10.0	4.6×10^{-2}	2.3×10^{-2}
Firing rate, I	44.3	45.9	11.3	12.0	43.5	45.0	0.37	0.32
FF, 5 ms, E	0.9585	0.9647	0.0148	0.0162	0.9576	0.9640	4.53×10^{-4}	1.12×10^{-4}
FF, 5 ms, I	0.8725	0.8726	0.0093	0.0091	0.8690	0.8688	9.4×10^{-4}	5.81×10^{-4}
FF, 100 ms, E	1.0573	1.0587	0.0345	0.0305	1.0493	1.0504	0.0074	0.0024
FF, 100 ms, I	1.1449	1.1540	0.0810	0.0859	1.1460	1.1528	0.0164	0.0099
ρ^{EE}, 5 ms ($\times 10^{-3}$)	1.9	2.0	1.5	1.1	1.9	2.1	1.5	1.0
ρ^{EE}, 50 ms ($\times 10^{-3}$)	5.8	6.3	6.2	4.8	6.0	6.4	5.9	4.6
ρ^{EE}, 100 ms ($\times 10^{-3}$)	5.9	6.3	7.5	5.3	5.9	6.4	7.2	5.3

Comparing Monte Carlo simulations with predictions from linear response; firing statistics in the asynchronous regime. Statistics displayed here are: firing rates for both excitatory and inhibitory populations; Fano factor (FF) for both excitatory and inhibitory populations; spike count correlations for excitatory pairs only (ρ^{EE}). Standard deviations are reported across the population; i.e. across eighty (80) E cells, or twenty (20) I cells, or 3160 E-E pairs.
