# Supplementary material for: When do correlations increase with firing rates in recurrent networks?
Source: PLoS Comput Biol. 2017 Apr 27;13(4):e1005506. doi: 10.1371/journal.pcbi.1005506 (PMC5426798; doi:10.1371/journal.pcbi.1005506)
Supplement: S3 Table — (PDF) [file pcbi.1005506.s013.pdf]

Table S3: **Statistics from heterogeneous vs. homogeneous networks: strong asynchronous regime**

	Heterogenous		Homogenous	
Statistic	E	I	E	I
Firing rate (Hz)	8.1 ± 4.5	36.6 ± 9.8	7.2 ± 0.095	35.2 ± 0.41
Var_T, $T = 5$ ms	0.039 ± 0.021	0.16 ± 0.040	0.035 ± 0.0004	0.15 ± 0.0016
Var_T, $T = 100$ ms	0.84 ± 0.48	3.93 ± 1.31	0.74 ± 0.013	3.75 ± 0.079
	Heterogenous		Homogenous	
ρ^{EE}, $T = 5$ ms	0.0119 ± 0.0037		0.0109 ± 0.0025	
ρ^{EE}, $T = 50$ ms	0.0622 ± 0.0206		0.0587 ± 0.0147	
ρ^{EE}, $T = 100$ ms	0.0654 ± 0.0232		0.0618 ± 0.0169	

Firing statistics from Monte Carlo simulations of recurrent networks in the strong asynchronous regime.
