# Supplementary material for: When do correlations increase with firing rates in recurrent networks?
Source: PLoS Comput Biol. 2017 Apr 27;13(4):e1005506. doi: 10.1371/journal.pcbi.1005506 (PMC5426798; doi:10.1371/journal.pcbi.1005506)
Supplement: S4 Table — (PDF) [file pcbi.1005506.s014.pdf]

Table S4: **Statistics in recurrent networks: Monte Carlo vs. linear response theory, strong asynchronous regime**

	Heterogenous				Homogenous			
	μ		σ		μ		σ	
Statistic	MC	LR	MC	LR	MC	LR	MC	LR
Firing rate, E	8.14	6.84	4.5	4.2	7.2	6.0	0.095	0.057
Firing rate, I	36.6	36.4	9.8	9.9	35.2	34.9	0.41	0.36
FF, 5 ms, E	0.9622	0.9829	0.0190	0.0155	0.9653	0.9853	4.80×10^{-4}	2.69×10^{-4}
FF, 5 ms, I	0.8719	0.8802	0.0147	0.0136	0.8709	0.8788	0.0014	0.0012
FF, 100 ms, E	1.0271	1.0578	0.0226	0.0206	1.0216	1.0516	0.0116	0.0059
FF, 100 ms, I	1.0581	1.0881	0.0698	0.0733	1.0655	1.0948	0.0124	0.0118
ρ^{EE}, 5 ms ($\times 10^{-3}$)	11.9	8.0	3.7	2.9	10.9	7.6	2.5	1.9
ρ^{EE}, 50 ms ($\times 10^{-3}$)	62.2	41.9	20.6	17.2	58.7	40.3	14.7	12.0
ρ^{EE}, 100 ms ($\times 10^{-3}$)	65.4	44.2	23.2	19.4	61.8	42.8	16.9	14.0

Comparing Monte Carlo simulations with predictions from linear response; firing statistics in the strong asynchronous regime. Statistics displayed here are: firing rates for both excitatory and inhibitory populations; Fano factor (FF) for both excitatory and inhibitory populations; spike count correlations for excitatory pairs only (ρ^{EE}). Standard deviations are reported across the population; i.e. across eighty (80) E cells, or twenty (20) I cells, or 3160 E-E pairs.
